# Synergistic Anti-Tumor Activity of EZH2 Inhibitors and Glucocorticoid Receptor Agonists in Models of Germinal Center Non-Hodgkin Lymphomas

**DOI:** 10.1371/journal.pone.0111840

**Published:** 2014-12-10

**Authors:** Sarah K. Knutson, Natalie M. Warholic, L. Danielle Johnston, Christine R. Klaus, Tim J. Wigle, Dorothy Iwanowicz, Bruce A. Littlefield, Margaret Porter-Scott, Jesse J. Smith, Mikel P. Moyer, Robert A. Copeland, Roy M. Pollock, Kevin W. Kuntz, Alejandra Raimondi, Heike Keilhack

**Affiliations:** 1 Research and Development, Epizyme Inc., Cambridge, Massachusetts, United States of America; 2 Oncology, Eisai Inc., Andover, Massachusetts, United States of America; University of Ulm, Germany

## Abstract

Patients with non-Hodgkin lymphoma (NHL) are treated today with a cocktail of drugs referred to as CHOP (Cyclophosphamide, Hydroxyldaunorubicin, Oncovin, and Prednisone). Subsets of patients with NHL of germinal center origin bear oncogenic mutations in the EZH2 histone methyltransferase. Clinical testing of the EZH2 inhibitor EPZ-6438 has recently begun in patients. We report here that combining EPZ-6438 with CHOP in preclinical cell culture and mouse models results in dramatic synergy for cell killing in *EZH2* mutant germinal center NHL cells. Surprisingly, we observe that much of this synergy is due to Prednisolone – a glucocorticoid receptor agonist (GRag) component of CHOP. Dramatic synergy was observed when EPZ-6438 is combined with Prednisolone alone, and a similar effect was observed with Dexamethasone, another GRag. Remarkably, the anti-proliferative effect of the EPZ-6438+GRag combination extends beyond EZH2 mutant-bearing cells to more generally impact germinal center NHL. These preclinical data reveal an unanticipated biological intersection between GR-mediated gene regulation and EZH2-mediated chromatin remodeling. The data also suggest the possibility of a significant and practical benefit of combining EZH2 inhibitors and GRag that warrants further investigation in a clinical setting.

## Introduction

Cellular differentiation, maturation and proliferation are all critically dependent on highly controlled programs of gene transcription [1]. Gene transcriptional responses depend on signal transduction pathways [2] in conjunction with a myriad of covalent modifications of chromatin components (e.g., site-specific methylation of histone proteins) [3], [4]. Our understanding of signal transduction and chromatin modification has been facilitated by interfacing the sciences of chemical biology and pharmacology [5], [6]. For example, the availability of ligands for components of nuclear hormone receptor signaling pathways, such as the glucocorticoid receptor (GR) pathway, has allowed scientists to divine the components and ordering of this pathway, and provided clinicians with invaluable therapeutics – in the form of GR agonists (GRag) – for the treatment of hyper-proliferative diseases [7]. Similarly, inhibitors of chromatin modifying enzymes are enhancing our understanding of this important mechanism of transcriptional control and are beginning to yield new therapeutic approaches for cancer [8]. There is a general acknowledgement that these molecular pathways must intersect at key points, but a detailed understanding of the connectivities between signal transduction and chromatin modification remains incomplete. In addressing best practices for the clinical use of our inhibitor (EPZ-6438 or E7438) of the chromatin-modifying enzyme EZH2 together with currently used drugs for NHL patients, we have identified an unexpected interplay between GR signal transduction and EZH2-mediated chromatin modification, which we report here.

Diffuse large B cell lymphoma (DLBCL) is subdivided into two groups: germinal center B-cell like (GCB) and activated B-cell like (ABC) [9], [10]. They can be distinguished by gene expression profiling or a sequence of immunohistochemical stainings (Hans-Choi algorithm) [11], [12]. CHOP (Cyclophosphamide, Hydroxyldaunomycin [Doxorubicin], Oncovin [Vincristine] and Prednisone), in combination with Rituximab (R-CHOP) is the current standard of care (SOC) for DLBCL [13], [14]. Recently, oncogenic mutations in *EZH2* – an enzyme that catalyzes methylation of the lysine 27 residue of histone H3 (H3K27) - have been found in a subset of GCB DLBCL patients [15], [16], [17]. Three hotspots were identified: Y646, A682 and A692 (referring to *EZH2* variant NM_004456.3). The recent development of potent and selective small molecule inhibitors of EZH2 has revealed that EZH2 mutant-bearing DLBCL cells are highly sensitive to EZH2 inhibition [18], [19], [20], [21], [22]. One such inhibitor (EPZ-6438) potently kills DLBCL cells bearing oncogenic mutations in *EZH2*, with minimal effect on the proliferation of wild-type *EZH2* DLBCL cells [23]; EPZ-6438 recently entered clinical testing as E7438 for patients with *EZH2* mutant NHL (NCT01897571). Here we demonstrate that the anti-proliferative effects of EPZ-6438 are greatly enhanced when combined with CHOP, and that most of this synergy can be ascribed to the GRag component of CHOP, Prednisolone (an active metabolite of Prednisone). Remarkably, the combination of EPZ-6438 and Prednisolone extends the range of cells that are sensitive to EZH2 inhibition, from the mutant bearing GCB type to include *EZH2* wild-type GCB NHL cells as well.

## Results

### EPZ-6438 shows combination benefit with lymphoma therapies in vitro

We investigated a possible combination benefit with EPZ-6438 and CHOP by pre-treating two *EZH2* mutant cell lines, WSU-DLCL2 and SUDHL10, with EPZ-6438 for 4 days, then co-treating with a combination of EPZ-6438 plus individual CHOP components for 3 additional days (4+3 model, materials and methods sections 1 and 2). A 4+3 model was chosen since H3K27Me3 inhibition by EPZ-6438 is maximal after 4 days with limited effects on lymphoma cell growth at that time point [23], while the agents of CHOP components have a faster effect on cell growth. Mafosfamide (a Cyclophosphamide analogue), Doxorubicin, and Vincristine all showed concentration-dependent growth inhibition in the mutant cell lines by themselves (S1 File table A). Therefore, combination indices (CI) were obtained for these drugs together with EPZ-6438. These cells, however, showed no sensitivity to Prednisolone alone. Hence, a CI could not be determined and instead an enhancement of potency was calculated based on the shift in IC_50_ of EPZ-6438 induced by varied concentration of Prednisolone. Equation A (in supplementary text in S1 File) describes the expected behavior when the IC_50_ for antiproliferative activity of one agent is affected by a second agent. The term α in this equation defines the degree of potency enhancement caused by the second agent and is the ratio of the IC_50_ values for the first agent in the presence over that in the absence of infinite concentration of the second agent (equation B in supplementary text in S1 File). The reciprocal of α (equation C in supplementary text in S1 File) provides an estimate of the maximum fold shift in IC_50_ of the first agent caused by the presence of the second agent (S1 File table B).

**Figure 1:**
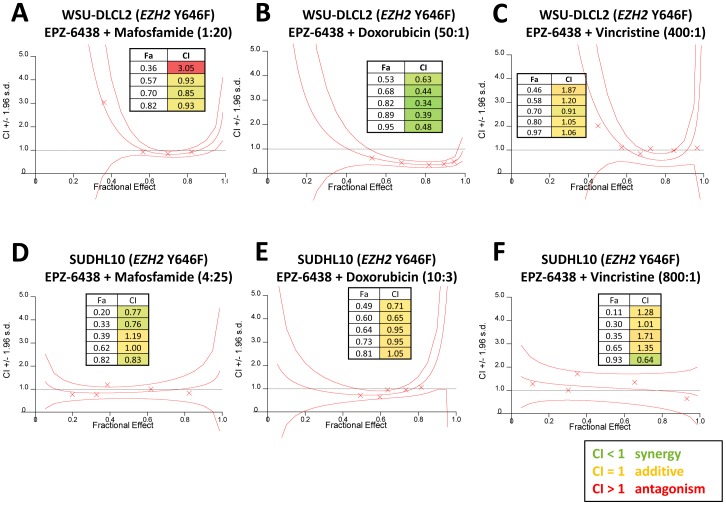
Combination benefit with CHOP components and EPZ-6438 in *EZH2* mutant germinal center B-cell lymphoma cell lines. Combination index (CI) graphs, generated in Calcusyn, of EPZ-6438 with Mafosfamide, Doxorubicin, or Vincristine in the *EZH2* Y646F mutant cell lines WSU-DLCL2 (A–C) or SUDHL10 (D–F). The 95% confidence interval is displayed in each graph (representative of 2 biological replicates for each cell line). The fractional effect (Fa) plotted is the fraction of cell growth (inhibition) resulting from a compound treatment, calculated from the DMSO control. A) Additivity was induced for the EPZ-6438/Mafosfamide combination at a 1∶20 constant ratio (doses were 16–125 nM for EPZ-6438 and 313–2500 nM for Mafosfamide). B) Synergy was induced for the EPZ-6438/Doxorubicin combination at a 50∶1 constant ratio (doses were 16–500 nM for EPZ-6438 and 0.3–10 nM for Doxorubicin). C) Additivity was induced for the EPZ-6438/Vincristine combination at a 400∶1 constant ratio (doses were 16–1000 nM for EPZ-6438 and 0.39–2.5 nM for Vincristine). D) Additivity was induced for the EPZ-6438/Mafosfamide combination at a 4∶25 constant ratio (doses were 12.5–200 nM for EPZ-6438 and 78–1250 nM for Mafosfamide). E) Additivity was induced for the EPZ-6438/Doxorubicin combination at a 10∶3 constant ratio (doses were 3–50 nM for EPZ-6438 and 0.94–15 nM for Doxorubicin). F) Additivity was shown for the EPZ-6438/Vincristine combination at an 800∶1 constant ratio (doses were 12.5–200 nM for EPZ-6438 and 15.6–250 pM for Vincristine).

**Figure 2:**
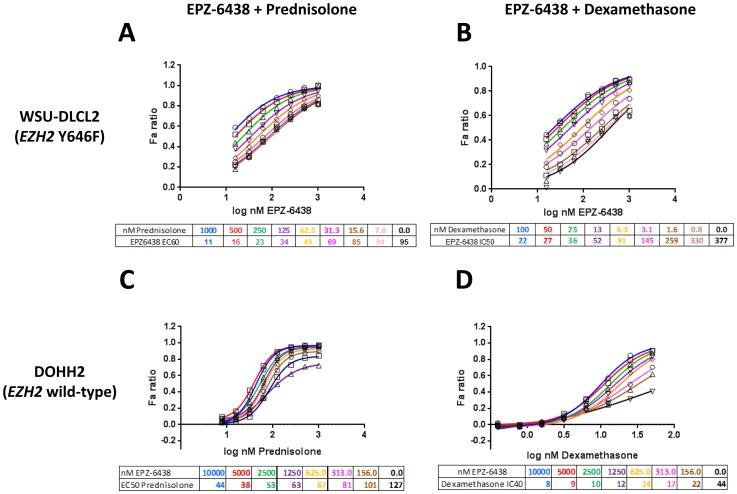
Glucocorticoid agonists enhance potency of EPZ-6438 in *EZH2* mutant and wild-type germinal center B cell lymphoma. Combinations of EPZ-6438 with Prednisolone or Dexamethasone in WSU-DLCL2 *EZH2* mutant (A, B) and DOHH2 *EZH2* wild-type (C, D) GCB lymphoma cell lines, respectively. All dose response plots were generated in Graphpad Prism and curves fitted to a four-parameter model with variable slope (2 biological replicates). Doses of EPZ-6438 ranged from 15.6–1000 nM, doses of Prednisolone ranged from 7.8–1000 nM, and doses of Dexamethasone ranged from 0.8–100 nM. A, B) Potency of EPZ-6438 was increased with Prednisolone or Dexamethasone in *EZH2* mutant WSU-DLCL2 cells. C, D) EPZ-6438 showed no anti-proliferative effect as a single agent in DOHH2 *EZH2* wild-type cells, therefore the potency shift of Prednisolone or Dexamethasone was measured. The potency of Prednisolone or Dexamethasone was increased with addition of EPZ-6438 in DOHH2 cells.

**Figure 3:**
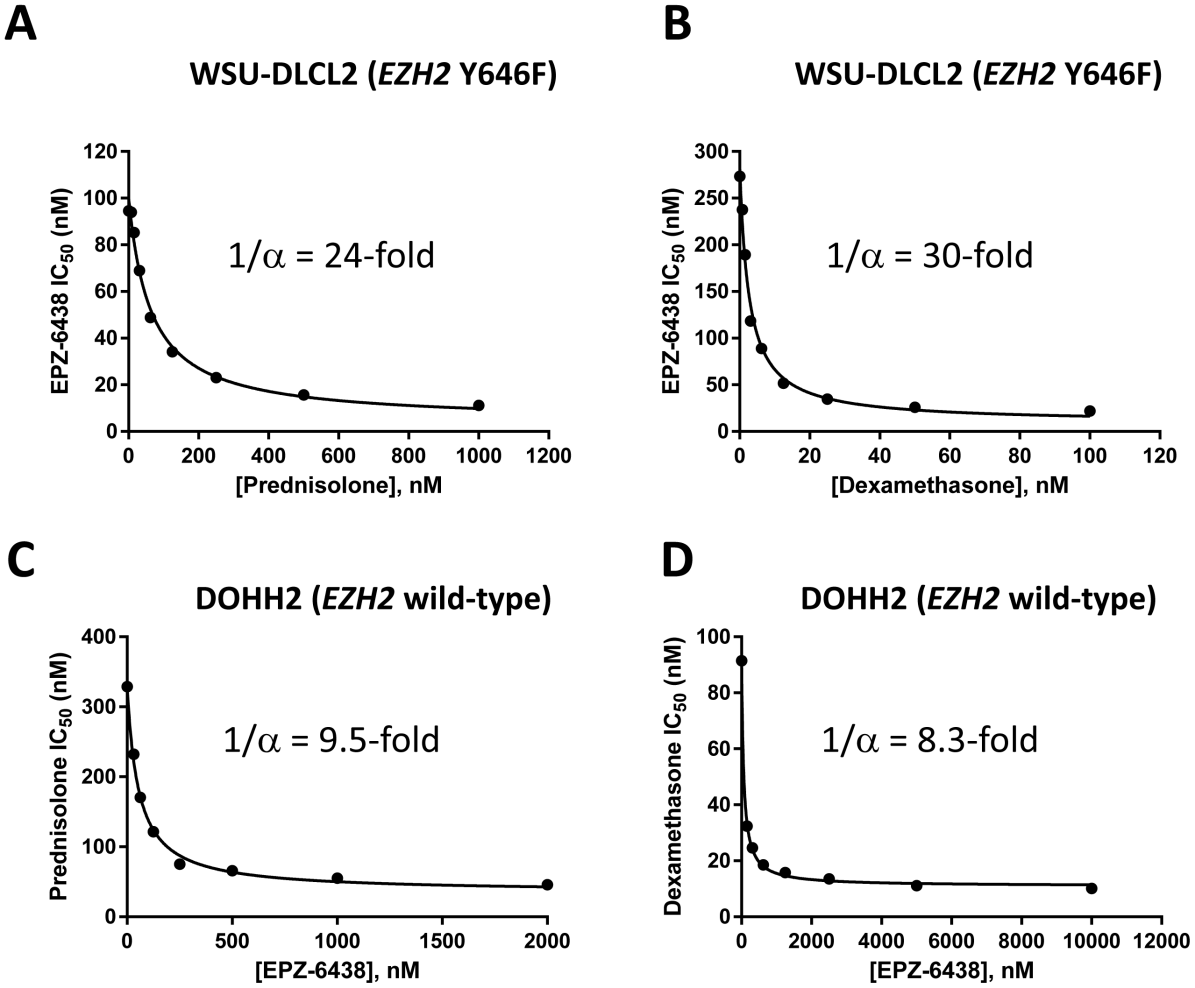
Modeling of the maximum combination effects between glucocorticoid receptor agonists and EPZ-6438 in *EZH2* mutant and wild-type germinal center B cell lymphoma. Combinations of EPZ-6438 with Prednisolone (A, C) or Dexamethasone (B, D) in WSU-DLCL2 *EZH2* mutant (A, B) and DOHH2 *EZH2* wild-type (C, D) GCB lymphoma cell lines. The data presented in Fig. 2 were analyzed such that a maximal possible shift in potency was calculated (1/α, see supplementary text in S1 File). A, B) Potency of EPZ-6438 was increased by a maximum 24-fold with Prednisolone and 30-fold with Dexamethasone in *EZH2* mutant WSU-DLCL2 cells. C, D) EPZ-6438 shows no anti-proliferative effect as a single agent in DOHH2 *EZH2* wild-type cells, therefore the potency shift of Prednisolone or Dexamethasone was measured. Potency of Prednisolone was increased a maximum of 9.5-fold with addition of EPZ-6438, and potency of Dexamethasone was increased a maximum of 8.3-fold with addition of EPZ-6438 in DOHH2 cells.

The combination of EPZ-6438+Mafosfamide displayed additivity in both *EZH2* mutant cell lines (Fig. 1A, D). In WSU-DLCL2 cells, EPZ-6438+Doxorubicin acted synergistically in the 4+3 model (Fig. 1B), while this combination was additive in SUDHL10 cells (Fig. 1E). The combination of EPZ-6438+Vincristine also demonstrated additivity in both cell lines (Fig. 1C, F). Treatment of WSU-DLCL2 cells with Prednisolone+EPZ-6438 caused an enhancement of EPZ-6438 activity (Fig. 2A), with a maximum 24-fold reduction in EPZ-6438 IC_50_ (Fig. 3A and S1 File table B). Treatment with a different GRag, Dexamethasone, resulted in an even greater 30-fold reduction in the IC_50_ of EPZ-6438 (Fig. 2B, 3B and S1 File table B). At biologically relevant concentrations of 1 µM for Prednisolone and 100 nM for Dexamethasone the potency enhancements were 7 and 15-fold, respectively (S1 File table C); enhancement of EPZ-6438 potency was also observed in SUDHL10 and SUDHL6 cells (S1 File figure A, tables B and C).

### EPZ-6438/GRag synergy is observed in GCB lymphoma cells independent of EZH2 mutation status

We next investigated if the combination effect of EPZ-6438+CHOP could render *EZH2* wild-type GCB lymphoma cell lines sensitive to EPZ-6438 (materials and methods section 1 and 2). To verify the germinal center origin on all cell lines tested we performed Hans-Choi immunohistochemistry on fixed cell pellets embedded in a solid matrix (S1 File table D and materials and methods section 3). Since EPZ-6438 treatment alone does not strongly inhibit growth in *EZH2* wild-type GCB lymphoma lines, we evaluated its ability to change the concentration-response curves of individual CHOP components. Interestingly, when tested in a wild-type GCB lymphoma cell line (DOHH2), only the GRag component of CHOP demonstrated enhanced potency in the presence of EPZ-6438 (Fig. 2C, D and 3C, D; S1 File tables B and C). In another wild-type GCB lymphoma line (Toledo) no potency shift was observed with any CHOP component (S1 File tables B and C). When we looked at two additional wild-type GCB lymphoma lines (SUDHL5 and OCI-LY19), the potencies of both Prednisolone and Dexamethasone were strongly enhanced by EPZ-6438 (S1 File tables B and C). In summary, we observed EPZ-6438/GRag combination benefit in three out of four *EZH2* wild-type GCB cell lines analyzed.

Given that only GRag+EPZ-6438 combinations induced dramatically enhanced anti-proliferative effects, compared to either single agent, in *EZH2* mutant and three out of four *EZH2* wild-type GCB lymphoma cell lines, we evaluated this combination in greater detail. Specifically, we wondered if duration of treatment and/or sequence of compound addition affected sensitivity (materials and methods section 1). We also expanded the analysis to include two additional *EZH2* mutant cells (RL, *EZH2* Y646N, and SUDHL4, *EZH2* Y646S). These *EZH2* mutant cells were chosen because, unlike the majority of *EZH2* mutant lines, they are insensitive to EZH2 inhibition for reasons that are not yet understood [19]. We had shown previously, however, that H3K37Me3 is inhibited by EPZ-6438 with similar potency in RL cells as in the sensitive *EZH2* mutant cell lines [23]. We therefore wished to determine whether these cells too would be rendered sensitive to the GRag+EPZ-6438 combination.

In the 4+3 model described above, the potency shift being measured was that of either EPZ-6438 (in *EZH2* Y646 mutant, sensitive cells) or Prednisolone (in *EZH2* wild-type cells). For these experiments, combination benefit was assessed as the change in EPZ-6438 IC_50_ at a fixed concentration of Prednisolone (materials and methods section 1). Cells were co-treated with both agents for 4 or 7 days, or treated with one agent for 4 days followed by 3 day co-treatment. When *EZH2* mutant, EPZ-6438 sensitive cells were co-treated for 4 days, a 30–60 fold lower IC_50_ of EPZ-6438 was observed in the presence of Prednisolone (table 1), demonstrating similar trends as observed above (Figs. 2A, B and 3 A, B; S1 File tables B and C). Similar results were observed with 7 day co-treatment and in the 4+3 model (table 1). In DOHH2 and OCI-LY19 *EZH2* wild-type GCB cells, decreased proliferation and measurable EPZ-6438 IC_50_ values were observed after 4 days of co-treatment with Prednisolone, despite a complete lack of EPZ-6438 single agent activity. *EZH2* wild-type GCB cells also responded to the 4+3 model and/or 7 day co-treatment schedules. Strikingly, *EZH2* mutant, EPZ-6438 insensitive cells, behaved much like the GCB *EZH2* wild-type cells, exhibiting a measurable EPZ-6438 proliferation IC_50_ after 4 day co-treatment with Prednisolone, despite a complete lack of sensitivity to EPZ-6438 alone. Treatment of the same cells under a 4+3 schedule or a 7 day co-treatment gave an even greater response (table 1). Interestingly, only 1 of the 6 cell lines (RL) demonstrated a significant combination benefit when cells were pre-treated with Prednisolone, then co-treated with EPZ-6438+Prednisolone, suggesting that the order of drug addition is important for the synergy effect.

**Table 1:** EPZ-6438/GRag combination increases EPZ-6438 sensitivity in *EZH2* Y646 mutant cell lines and overcomes insensitivity in cell lines resistant to EZH2 inhibition.

Cell Line/*EZH2* Status/EZH2 inhibitor sensitivity	Pred IC_50_ Day 3 (µM)	EPZ-6438 IC_50_, µM, Day 4		EPZ-6438 IC_50_, µM, Day 7		
		EPZ-6438 alone	EPZ-6438/Pred co-treatment	4-day EPZ-6438 = >3-day EPZ-6438/Pred	4-day Pred = >3-day EPZ-6438/Pred	7-day EPZ-6438/Pred
WSU-DLCL2 *EZH2* Y646 sensitive	>1	0.53±0.014	0.02±0.021	0.011±0.0062	>1	0.014±0.0049
SUDHL10 *EZH2* Y646F sensitive	>1	0.64±0.26	0.0092±0.0044	0.0027±0.0013	0.52,>1	0.02±0.0057
RL *EZH2* Y646N insensitive	>1	>1	0.0096±0.0066	>>0.004	0.38	>0.004
SUDHL4 *EZH2* Y646S insensitive	>1	>1	>1, 0.2,>1	0.035±0.043	>1	0.51±0.35
DOHH2 *EZH2* wild-type insensitive	1.3	>1	0.2±0.25	>1, 0.03,>1	>1	0.34±0.078
OCI-LY19 *EZH2* wild-type insensitive	0.059	>1	0.19±0.11	0.0055±0.0047	>1	0.026, <0.004

Values in the table represent EPZ-6438 mean IC_50_ values ± SEM (at least n = 2) for the indicated time points and dosing schedules. EPZ-6438 IC_50_ values were not extrapolated beyond the highest and lowest doses of EPZ-6438 (1 µM, and 0.004 µM, respectively). In cases where IC_50_ values could not be averaged due to non-extrapolated values, all replicates are listed. GRag: glucocorticoid receptor agonist, Pred: Prednisolone.

### Global H3K27 acetylation or trimethylation is not altered in the EPZ-6438/GRag combination, compared to single agent treatments

To evaluate potential mechanisms responsible for the observed combination benefits of EPZ-6438+GRag in these cell lines, we determined whether Prednisolone treatment affected global methylation and acetylation of H3K27 following a four day treatment either alone or in combination with EPZ-6438 in WSU-DLCL2, OCI-LY19, and RL cells (two independent experiments, materials and methods section 4). Single agent Prednisolone had no effect on H3K27Me3 levels in WSU-DLCL2 or RL cells, but did increase H3K27Me3 levels at higher doses in OCI-LY19 cells (S1 File figure B). Due to the high sensitivity of OCI-LY19 cells to Prednisolone, in contrast to the Prednisolone-insensitive EZH2 mutant lines (S1 File table A), a lower Prednisolone dose was necessary for the treatment of OCY-LY19 cells. The inclusion of Prednisolone did not alter the EPZ-6438 IC_50_ for H3K27Me3 inhibition in any cell line (S1 File figure B). Likewise, global H3K27 acetylation levels were not affected by Prednisolone alone or in combination with EPZ-6438 (S1 File figure C).

### EPZ-6438/GRag combination synergistically influences gene expression

**Figure 4:**
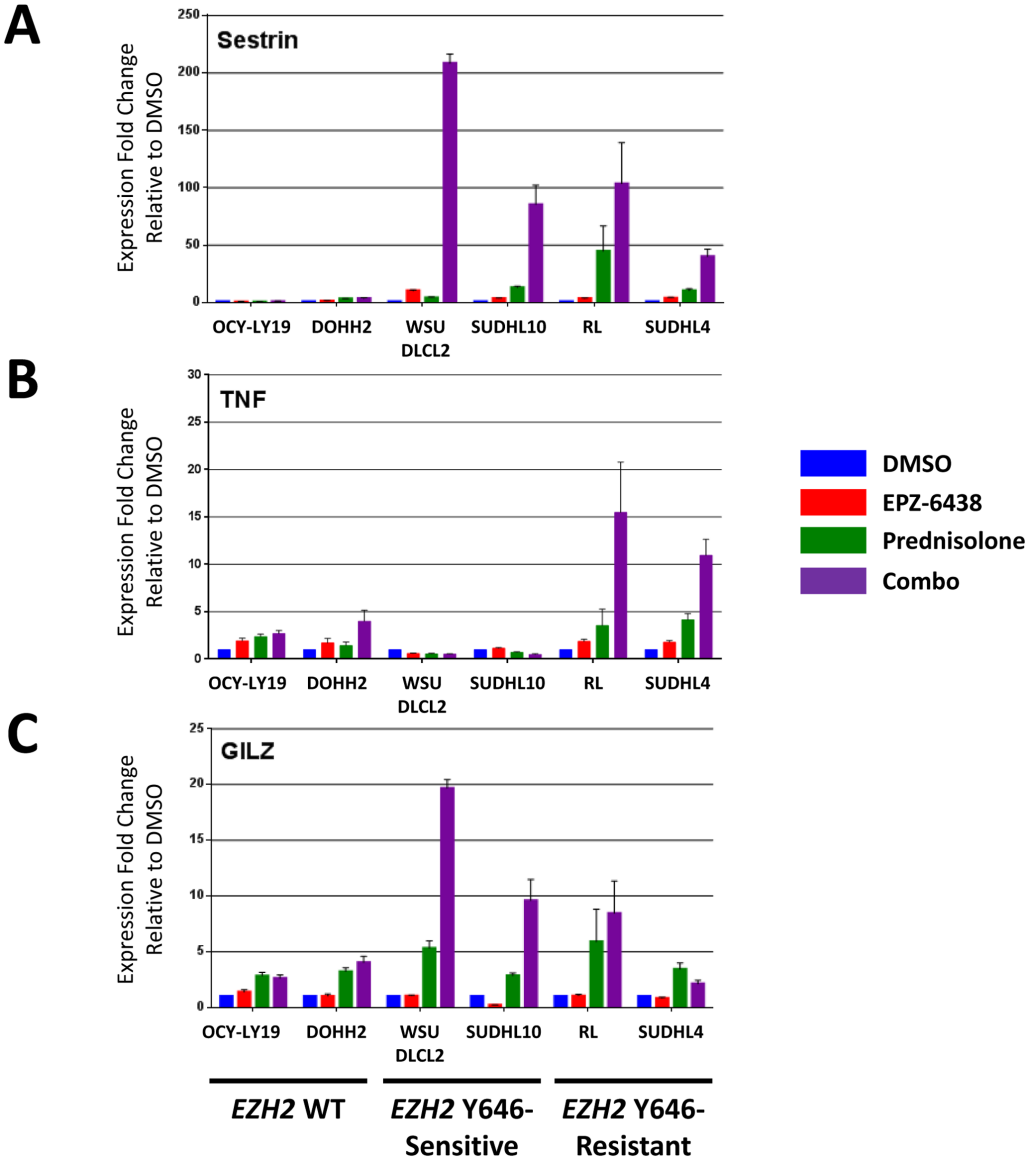
Glucocorticoid target genes are up-regulated by prednisolone/EPZ-6438 combination in *EZH2* mutant cell lines. Expression levels of Sestrin 1 (*SESN1*, A), *TNF* (B) and *GILZ* (C), normalized to DMSO controls, for each cell line treated with the indicated single agents or their combination (2 biological replicates, see materials and methods section 5 for details). Fold change values were quantified using the ΔΔCt method, and *ACTB*, *B2M* and *GAPDH* as reference genes. Error bars represent SEM values. Statistical analysis of the data is presented in table 2. WT: wild-type.

Having found no significant combination-specific effects on global levels of H3K27 acetylation or trimethylation, we next looked directly at transcriptional regulation of GR signaling pathways. WSU-DLCL2, SUDHL10, RL, SUDHL4, OCI-LY19, and DOHH2 cells were treated with a single concentration of EPZ-6438, Prednisolone, or their combination for 4 days, and gene expression was analyzed using a glucocorticoid signaling PCR array (materials and methods section 5; S2 File). A larger number of genes were down-regulated with both Prednisolone and combination treatments in all cells, pointing to a role of GR as both activator and repressor of transcription [24]. Here, we focus on the activating function of GR and describe 3 genes which show synergistic up-regulation upon combination treatment. Sestrin 1 (*SESN1*), a putative tumor suppressor and mTOR signaling inhibitor [25], was identified as a gene synergistically up-regulated commonly in 3 of 4 *EZH2* mutant cells with combination treatment, but not in *EZH2* wild-type cells (Fig. 4A and table 2). Interestingly, *TNF* expression was synergistically up-regulated in a statistical significant manner only in one of the two *EZH2* mutant, EPZ-6438 insensitive cell lines (SUDHL4), with a trend for the other *EZH2* mutant, EPZ-6438 insensitive cell line (RL) showing the same result (Fig. 4B and table 2). Expression of *TSC22D3/GILZ*, while up-regulated in all cell lines by Prednisolone, is only synergistically enhanced by combination treatment in *EZH2* mutant, EPZ-6438 sensitive cells (Fig. 4C and table 2). Other classic GR regulated genes, or GR itself, were not commonly affected among cell lines in the combination (S2 File; S1 File table E and figure D).

**Table 2:** Statistical analysis of gene expression data presented in Fig. 4.

Cell Line	Comparison	*Sestrin*		*TNF*		*GILZ*	
		*P* Value	*P* Value Summary	*P* Value	*P* Value Summary	*P* Value	*P* Value Summary
OCI-LY19	DMSO vs Combo	0.9164	ns	0.0071	**	0.0075	**
OCI-LY19	EPZ-6438 vs Combo	0.3232	ns	0.1553	ns	0.0326	*
OCI-LY19	Prednisolone vs Combo	0.1486	ns	0.5050	ns	0.6353	ns
DOHH2	DMSO vs Combo	0.0063	**	0.0589	ns	0.0056	**
DOHH2	EPZ-6438 vs Combo	0.0186	*	0.1401	ns	0.0071	**
DOHH2	Prednisolone vs Combo	0.557	ns	0.1000	ns	0.2828	ns
WSU-DLCL2	DMSO vs Combo	<0.0001	****	0.0001	***	<0.0001	****
WSU-DLCL2	EPZ-6438 vs Combo	<0.0001	****	0.3813	ns	<0.0001	****
WSU-DLCL2	Prednisolone vs Combo	<0.0001	****	0.9483	ns	0.0001	***
SUDHL10	DMSO vs Combo	0.0073	**	0.0058	**	0.0102	*
SUDHL10	EPZ-6438 vs Combo	0.0081	**	0.0050	**	0.0076	**
SUDHL10	Prednisolone vs Combo	0.0126	*	0.1159	ns	0.0236	*
RL	DMSO vs Combo	0.0449	*	0.0529	ns	0.0623	ns
RL	EPZ-6438 vs Combo	0.0484	*	0.0639	ns	0.0635	ns
RL	Prednisolone vs Combo	0.2329	ns	0.0997	ns	0.5716	ns
SUDHL4	DMSO vs Combo	0.0033	**	0.0043	**	0.0275	*
SUDHL4	EPZ-6438 vs Combo	0.0045	**	0.0059	**	0.0196	*
SUDHL4	Prednisolone vs Combo	0.010	*	0.0205	*	0.0107	ns

Pairwise statistical comparisons were performed by two-tailed *t* test.

ns: not significant;

* *p*<0.05;

** *p*<0.01;

*** *p*<0.001;

**** *p*<0.0001.

### EPZ-6438 combinations with CHOP or Prednisone enhance anti-tumor activity in vivo, in comparison to single agent dosing

**Figure 5:**
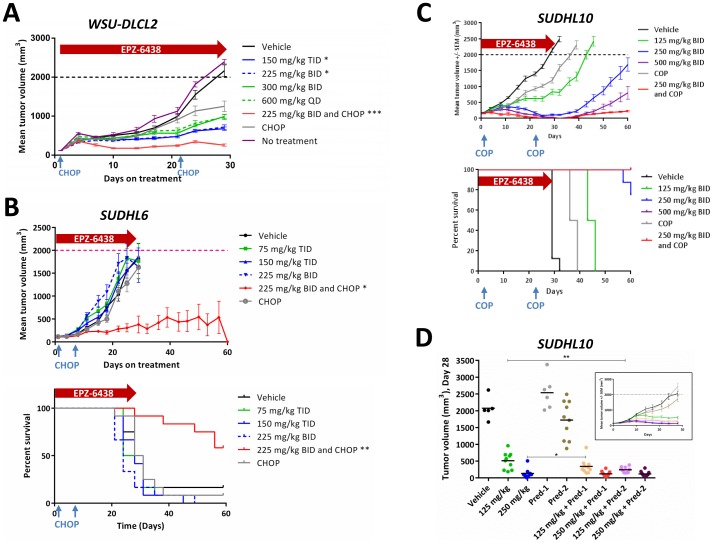
EPZ-6438/CHOP combinations show enhanced anti-tumor activity compared to single agents in several *EZH2* mutant lymphoma xenograft models. A) WSU-DLCL2 (*EZH2* Y646F) xenograft-bearing mice were treated for 28 days as indicated. Mean tumor volumes ± SEM (n = 12) are plotted. Treatment with EPZ-6438 at 225 mg/kg BID plus CHOP induced the highest tumor growth inhibition (93%). B) SUDHL6 (*EZH2* Y646N) xenograft-bearing mice were treated for 28 days as indicated. Mean tumor volumes ± SEM (n = 12) are plotted in the top panel. * *p*<0.05, *** *p*<0.001 vs. vehicle, repeated measures ANOVA, Dunnett's post test. Kaplan-Meyer survival curves (bottom panel) out to 60 days demonstrate significant tumor growth delay in animals treated with EPZ-6438+CHOP (** *p*<0.01). C) SUDHL10 (*EZH2* Y646F) xenograft-bearing mice were treated with EPZ-6438, COP (chemotherapy without the Doxorubicin component), or their combination for 28 days. Mean tumor volumes ± SEM (n = 8) are plotted in top panel. Percent survival out to 60 days is plotted in the bottom panel (note: 500 mg/kg and 250 mg/kg+COP survival curves overlap). D) SUDHL10 (*EZH2* Y646F) xenograft-bearing mice were treated for 28 days as indicated (Pred-1 = Prednisone at 0.15 mg/kg BID×5 on days 1–5 and 22–26; Pred-2 = Prednisone at 0.15 mg/kg BID×28). The scatter blot shows the tumor volumes on day 28, and the inset shows the mean tumor volumes ±SEM (n = 10) followed over 28 days (also presented in figure S6B). * *p*<0.05, ** *p*<0.01; two-tailed *t* test. All groups administered EPZ-6438 show statistically significant smaller tumor volumes on day 28 (*p*<0.01 at least, vs. vehicle or Prednisone single agent at both schedules; two-tailed *t* test). CHOP: Cyclophosphamide, Hydroxyldaunorubicin (Doxorubine), Oncovin (Vincristine), Prednisone; COP: Cyclophosphamide, Oncovin (Vincristine), Prednisone; BID: two times a day every 12 hours; QD: once a day; TID: three times a day every 8 hours.

Finally, tumor growth inhibition was assessed in 3 different *EZH2* mutant lymphoma xenograft models (materials and methods section 6). SCID or nude mice bearing subcutaneous lymphoma xenografts were co-dosed with EPZ-6438 plus either CHOP or COP (CHOP without Doxorubicin), and compared to single agent treatments. In WSU-DLCL2 xenograft bearing mice, tumor growth inhibition (TGI) was achieved at all EPZ-6438 doses and schedules employed, and was better than CHOP alone (Fig. 5A). Moreover, the combination of EPZ-6438 and CHOP induced robust anti-tumor responses and significantly (*p*<0.001) better TGI (93%) than did either single agent (45% and 71%, for CHOP and EPZ-6438, respectively). All treatments were well tolerated; there was minor body weight loss (11.3%) in the EPZ-6438/CHOP combination group during the first cycle, after which mice recovered before the next cycle of treatment (S1 File figure E, panel A).

In SUDHL6 xenografts, significant TGI was not observed with CHOP or EPZ-6438 alone (Fig. 5B, top panel), in contrast to results previously published using the EZH2 inhibitor GSK503 [26]. Strikingly, the combination of EPZ-6438/CHOP resulted in tumor regression. When dosing was stopped on day 28 and mice were observed through day 60, this combination resulted in tumor free survival in 58% of the mice (Fig. 5B, lower panel). All treatments were well tolerated without loss of body weight (S1 File figure E, panel B).

Our in vitro studies suggested that Prednisone may be the key component of driving the in vivo combination benefit observed with the EPZ-6438/CHOP treatment. Hence, we studied the effects of omitting one or all chemotherapy components from the CHOP regime in two additional xenograft studies. First, we investigated the combination benefit of an EPZ-6438/chemotherapy regimen that eliminated Doxorubicin, which has a lifetime cumulative dosing limit of <550 mg/m^2^ due to its cardiotoxicity [27]. In SUDHL10 xenograft bearing mice, TGI was observed at all EPZ-6438 doses (Fig. 5C, top panel) as well as with Doxorubicin-free CHOP (COP). The 250 mg/kg EPZ-6438, 500 mg/kg EPZ-6438 and EPZ-6438/COP combination treatments resulted in regressions that were statistically different from vehicle (*p*<0.001), with the EPZ-6438/COP combination demonstrating the best overall response. When dosing was stopped on day 28, a clear dose dependent tumor growth delay benefit for EPZ-6438-treated mice was observed; COP treated tumors progressed faster than those treated with EPZ-6438 (Fig. 5C, lower panel). While mice dosed with the maximal tolerated dose of EPZ-6438 or with the EPZ-6438/COP combination showed 100% survival on day 60, the combination group showed the smallest day 28 tumor weights, statistically different (*p*<0.05) from all other treatment groups, including the maximal tolerated dose for EPZ-6438 (S1 File figure F, panel A). Finally, we investigated combination dosing of EPZ-6438 with Prednisone for 28 days in the SUDHL10 xenograft model. As suggested by the in vitro data, Prednisone dosing alone did not induce any significant anti-tumor effect (Fig. 5D and S1 File figure F, panel B). In line with the previous study, 125 mg/kg BID (twice daily) dosing of EPZ-6438 generated only a partial response, but co-dosing of EPZ-6438 with Prednisone at 0.15 mg/kg BID, but not with the 2 cycle Prednisone regimen, induced the maximal possible regression achieved with higher doses of EPZ-6438 alone.

## Discussion

Standard treatments for B-cell NHL are combination chemotherapy regimens composed of Cyclophosphamide, Doxorubicin, Vincristine and Prednisolone [13], [14]. While complete response rates of 40–50% can be achieved, approximately one third of patients relapse with 3-year overall survival rates of only about 30% [28]. Relapsed lymphomas can exhibit resistance to a wide range of anti-cancer drugs, which presents a severe clinical challenge in managing these aggressive malignancies. Acquisition of drug resistance in lymphoma is partly driven by the genetic heterogeneity and instability of the tumor cells. Successful treatment of chemoresistant NHL will thus require rational combinations of drugs, targeting multiple pathways specific to the different subtypes of B-cell NHL.

EZH2 is a critical oncogenic driver in germinal center-derived B-cell lymphomas [26]. These more primitive B-cell malignancies, especially variants expressing EZH2 mutants with altered activity require EZH2 for proliferation and survival. Results from preclinical studies forecast great promise for inhibitors of EZH2 catalysis as treatment for such genetically defined cancers [18], [19], [20], [21], [22], and EZH2 inhibitors may also mitigate chemotherapy resistance. Our data show that EPZ-6438, an early clinical stage EZH2 inhibitor, shows various degrees of combination benefit in preclinical models of GCB lymphoma, ranging from additivity to synergy with the components of CHOP. In the case of Cyclophosphamide, Doxorubicin and Vincristine, those combination effects in vitro were restricted to EZH2 mutant-bearing cells. In vivo, significant synergy in lymphoma cell killing was also found when EPZ-6438 was co-dosed with CHOP. This was especially true in the SUDHL6 xenograft model where neither single agent showed any significant anti-tumor activity, but the combination induced durable regressions in >50% of mice. This highlights the potential importance of overactive EZH2 in chemoresistance of *EZH2* mutant lymphomas.

Among the CHOP components, EPZ-6438 combinations with Prednisolone induced the strongest anti-proliferative activity, and this combination could also render refractory GCB lymphoma cell lines sensitive to EZH2 inhibition regardless of the *EZH2* mutational status. The Toledo cell line was an outlier within the GCB models investigated as no combination benefit was observed with any CHOP components. The reasons for that are unknown, but may be explained by the existence of other driver pathways besides EZH2 being active in this cell line, such as Myc [29]. Also, although our Hans-Choi immunohistochemistry data suggest that the Toledo cell line is of GCB origin (S1 File table D), the cell line has previously been described as either GCB or type 3 (uncategorized) in the literature [29], [30], questioning the precise cell origin for this line.

This surprising finding of the EPZ-6438/GRag synergy has potentially important implications for the application of EZH2 inhibitors in the clinic and should be further investigated in clinical studies. GRags are frequently co-administrated with anti-cancer drugs to prevent drug-induced allergic reactions, to relieve pain, nausea, and emesis [31], [32], and are pivotal in the treatment of hematopoietic malignancies owing to their ability to induce apoptosis in these cancers [33], [34]. Compared to the other CHOP components GRag induce the least severe adverse effects. Further, the opportunity to eliminate Doxorubicin or all chemotherapy components from CHOP, while preserving a combination benefit with EPZ-6438, as suggested by our data in the SUDHL10 xenograft model, could spare patients from severe chemotherapy side effects. Finally, preclinical studies have shown that single agent EZH2 inhibitors induce significant cell killing only in EZH2 mutant-bearing lymphomas, which represent a fraction (20%) of GCB DLBCL patients [15] with high unmet clinical need. Our results provide a basis for investigating whether GRag/EZH2 inhibitor combinations may have clinical utility in all germinal center derived B cell lymphomas.

Glucocorticoid-bound GR moves to the nucleus and acts as either a transcriptional activator or repressor, depending on the cellular environment [24]. It has been suggested that GR constantly samples the nucleosome for a productive interaction, and the purpose of chromatin-modifying enzymes is to provide regulated access of GR, its cofactors, and the basal transcription machinery to DNA [35]. Other studies show that GR often binds to regions of open chromatin, and the chromatin architecture in a given cell type is organized such that GR can act in a tissue-specific manner [36]. Thus, it is conceivable that aberrant chromatin repression, induced by EZH2 mediated hypertrimethylation of H3K27, can block some of the otherwise accessible GR binding sites, interfering with normal GR function. Indeed, *EZH2* mutant lymphoma cell lines are insensitive to GRag treatment, while concentration-dependent cell killing is observed in *EZH2* wild-type cells. Our observation that synergy is not observed when cells are treated with Prednisolone prior to the addition of EPZ-6438, in most cells tested, suggests that EZH2 inhibitor-induced chromatin remodeling may be a rate limiting step for the enhanced action of GRag.

GR pathway gene expression arrays revealed both increased and decreased expression after treatment of several GCB lymphoma cells (both *EZH2* wild-type and mutant) with either EPZ-6438, Prednisolone or their combination, confirming the dual function of GR. The only gene that was synergistically up-regulated in combination for nearly all *EZH2* mutant lymphoma cells (3 out of 4) was Sestrin 1 (*SESN1*), a TP53 tumor suppressor with functions in cellular response to DNA damage and oxidative stress. Sestrins inhibit cell growth by activating AMP-activated protein kinase, resulting in mTOR pathway inhibition [25]. Hence SESN1-mediated mTOR pathway inhibition may be an important mechanism of reintroducing GRag sensitivity in *EZH2* mutant lymphoma cells after EPZ-6438 treatment.

Conversely, GRag/EPZ-6438 combination treatment also induced cell killing in those *EZH2* mutant lymphoma cell lines that have been reported as refractory to EZH2 inhibitor treatment (RL, SUDHL4). Synergistic up-regulation of TNF, a potent inflammatory cytokine, was observed specifically in SUDHL4 cells, and the same trend, although not statistically significant, was observed in RL cells. This seems surprising as TNF and glucocorticoids usually act antagonistically [37]. TNF, through its receptor TNFR-1, can induce apoptosis, but also has the ability to transduce survival signals, mainly through the NFκB pathway [38]. It is thus possible that the action of the up-regulated TNF is shifted towards apoptosis in the context of the combination because GRag mediates trans-repression of NFκB-dependent transcription. It is unclear, however, why this mechanism would result in synergistic cell killing only in the EZH2 inhibitor insensitive *EZH2* mutant cells. The potential importance of aberrant repression of negative regulators of the NFκB pathway in GRag resistance and a potential role for EZH2 in this process is further supported by our observation that GILZ [39] is synergistically up-regulated in 2 out of 6 cells lines with the combination. While single cell lines showed significant expression changes in GR regulated genes we were unable to find common synergistic expression changes of many classic GR regulated genes or determine a common signaling pathway among all 6 cell lines which would identify a general mechanism of synergistic killing with GRag/EPZ-6438 treatment in GCB lymphoma. This may be explained by the inherent heterogeneity of cultured lymphoma cells, and is subject of ongoing broader investigations.

Taken together, our data reveal an unforeseen biological interaction between GR-regulated transcription and EZH2-mediated chromatin remodeling, which suggest potential new avenues for clinical investigation in patients with therapy resistant NHL.

## Materials and Methods

### 1) Cell lines, compounds, and treatment outline

Lymphoma cell lines OCI-LY19 (ACC-528), WSU-DLCL2 (ACC-575), SUDHL5 (ACC-571) and SUDHL10 (ACC-576) were obtained from DSMZ. RL (CRL-2261), Toledo (CRL-2631), SUDHL6 (CRL-2959) cells were obtained from ATCC. DOHH2 (HTL99022) was obtained from BBCF. Toledo and SUDHL6 cell lines were cultured in RPMI+20% FBS (fetal bovine serum), while all other cell lines were cultured in RPMI+10% FBS. All cell cultures were performed in full serum (not charcoal stripped), to better simulate physiological conditions and because growth conditions (growth curves, proliferation rates etc.) were not optimized for charcoal stripped serum. Cell lines were authenticated by short tandem repeat (STR) assay and EZH2 mutational status was verified by sequence analysis. For combination studies, a modified version of a previously published proliferation assay in suspension cells was used [40]. Briefly, on day 0, cells were plated in triplicate in 96-well plates at initial densities to ensure linear logarithmic phase growth over 4 days. Cells were treated with either a serial dilution of EPZ-6438 (starting at a top dose of 1 µM), a single dose of Prednisolone (Selleck Chemicals, #S1737) at a concentration 10-fold lower than the 4-day IC_50_ of the drug, or a combination of EPZ-6438+Prednisolone. On day 4, cells were counted using Viacount reagent in the Guava easyCyte flow cytometer, and the viable cell number was used to replate cells at the original densities for 3 additional days. Cells that were pre-treated with EPZ-6438 either received continuous EPZ-6438 alone, or EPZ-6438+Prednisolone (constant dose); cells pre-treated with Prednisolone either received continuous Prednisolone, or Prednisolone+EPZ-6438; cells co-treated for 4 days continued to receive co-treatment through 7 days.

### 2) Medium throughput assay

Compounds were purchased from commercial vendors (Mafosfamide-Santa Cruz SC-211761; Dexamethasone-Tocris 1126; Vincristine- Tocris 1257; Doxorubicin-Sigma 1515). Lymphoma cells were seeded into flasks (50,000 cells/mL for WSU-DLCL2, SUDHL5 and DOHH2; 10,000 cells/mL for SUDHL10; and 100,000 cells/mL for OCI-LY19 and Toledo) and pretreated with 7 doses of EPZ-6438 or DMSO for 4 days or 6 days for Toledo assays. Cells were then split back to 50,000 cells/mL for WSU-DLCL2, SU-DHL-5 and DOHH2 or 30,000 cells/mL for SUDHL10, and 100,000 for OCI-LY19 and Toledo, and then co-treated with EPZ-6438 and compound of interest using the HP D300 digital dispenser (Tecan). Both drugs were serially diluted two-fold and combined in a matrix with constant ratios diagonally across the plate with a final DMSO content of 0.11% (v/v). After 3 days of co-treatment (5 days for Toledo assays), cell viability was measured via ATP content using CellTiter-Glo (Promega) and luminescence was detected using a SpectraMax M5 microplate reader (Molecular Devices).

Synergy quantification was performed using the Chou-Talalay method for drug combination. The Combination Index (CI) equation offers a quantitative definition for additivity (CI = 1), synergism (CI<1), and antagonism (CI>1). This equation uses fractional effect (Fa) values (fraction of cell growth calculated from the DMSO control) from a constant ratio of drug combination to determine CI values. The resulting plot (Fa-CI) plot shows the resultant CI values bracketed by 95% confidence intervals. These Fa-CI plots are generated using Calcusyn for Windows software. CI values <1 with confidence interval lines also below 1 indicate statistically significant synergism.

When one of the combined drugs did not have an IC_50_, the extent of the combination benefit was determined by maximal fold shift in the IC_50_ of the other agent. The α constant and its reciprocal, 1/α were calculated to quantitate further the combinatorial effect as maximal fold IC_50_ shift (equations for α and its reciprocal value are described in the supplementary text in S1 File, equations B and C) [41].

### 3) Hans-Choi immunohistochemistry

Cultured lymphoma cell lines were fixed overnight at room temperature in 10% neutral buffered formalin and then embedded into paraffin blocks. Sections of 5 µM thickness were cut. Sections were deparaffinized and blocked with hydrogen peroxide, followed by antigen retrieval through steam treatment in Tris-EDTA buffer (pH 9) for 20 minutes. Slides were washed with PBST and then blocked with UV light for 5 minutes. Antibody dilutions (in Tris-BSA buffer) for immunohistochemistry were as follows: anti-BCL6 (Dako, clone PG-B6p, #M7211) 1∶400; anti-CD10 (Leica, clone 56C6, #CD10-270-CE-S) 1∶50; anti-FOXP1 (Cell Signaling # 2005S) 1∶200; anti-GCET1 (Abcam, clone RAM341, #ab6889) 1∶100; anti-MUM1 (Dako, clone MUM1p, #M7259), 1∶1,500. Staining with the primary antibodies was for 30 minutes, followed UV antibody enhancer for 10 minutes and UV polymer treatment for 15 minutes. Slides were incubated with DAB substrate solution for 10 minutes and counterstained with Hematoxylin for 5 minutes. The slides were coversliped and scored by a hematopathologist (results in S1 File table D).

### 4) ELISA and western blots

WSU-DLCL2, RL, and OCI-LY19 cells were treated in parallel with DMSO, 1 µM of EPZ-6438, a dose of Prednisolone at a concentration 10-fold lower than the 4-day IC_50_, or the combination of drugs for 4 days. Histones preparations were generated by acid extractions as previously described [40]. ELISA and western blots were carried out as previously described [18], [40], [42]. For the ELISA, histones were prepared in equivalent concentrations in coating buffer (PBS+0.05% bovine serum albumin [BSA]) yielding 0.5 ng/µl of sample, and 100 ul of sample or standard was added in duplicate to two 96-well ELISA plates (Thermo Labsystems, Immulon 4HBX #3885). The plates were sealed and incubated overnight at 4°C. The following day, plates were washed 3x with 300 µl/well PBST (PBS+0.05% Tween 20; 10X PBST, KPL #51-14-02) on a Bio Tek plate washer. Plates were blocked with 300 µl/well of diluent (PBS+2% BSA+0.05% Tween 20), incubated at room temperature (RT) for 2 hours, and washed 3x with PBST. All antibodies were diluted in diluent. 100 µl/well of anti-H3K27Me3 (CST #9733, 50% glycerol stock 1∶1,000) or anti-total H3 (Abcam ab1791, 50% glycerol 1∶10,000) were added to each plate. Plates were incubated for 90 min at RT and washed 3x with PBST. 100 µl/well of anti-Rb-IgG-HRP (Cell Signaling Technology, 7074) was added 1∶2,000 to the H3K27Me3 plate and 1∶6,000 to the H3 plate and incubated for 90 min at RT. Plates were washed 4x with PBST. For detection, 100 µl/well of TMB substrate (BioFx Laboratories, #TMBS) was added and plates incubated in the dark at RT for 5 min. Reaction was stopped with 100 µl/well 1N H_2_SO_4_. Absorbance at 450 nm was read on SpectaMax M5 Microplate reader. For the western blots, protein concentrations for acid extracted histones were determined by BCA assay (Pierce). 800 ng of each lysate was fractionated on 10–20% Tris-Glycine gel (Biorad), transferred using iBlot (7 minutes on program 3, using Nitrocellulose transfer stacks), and probed with the following antibodies in Odyssey blocking buffer: rabbit anti-H3K27acetyl (Active Motif 39133; 1∶1,000) and mouse anti-Total H3 (CST 3638; 1∶20,000 dilution). Following primary antibody incubation, membranes were probed with IRDye 800CW Donkey-anti-mouse IgG (LiCOR #926-32212) or Alexa Fluor 680 goat-anti-rabbit IgG (Invitrogen #A-21076) secondary Ab. Blots were imaged and analyzed using the LiCOR Odyssey system.

### 5) Quantitative PCR

WSU-DLCL2, SUDHL10, RL, SUDHL4, OCI-LY19, and DOHH2 cells were treated in parallel with DMSO, 1 µM of EPZ-6438 (SUDHL10 treated with 100 nM EPZ-6438), a dose of Prednisolone at a concentration 10-fold lower than the 4-day IC_50_, or the combination of drugs for 4 days. Cells were harvested and total mRNA was extracted from cell pellets using the RNeasy Plus Mini Kit (Qiagen; 74134). For the RT2 Glucocorticoid Signaling PCR array (Qiagen; PAHS-154ZE-4), cDNA was made by RT2 First Strand Kit (Qiagen; 330401). Array RT-PCR was performed using ViiA 7 Real-Time PCR Systems (Applied Biosystems [AB]) with RT2 SYBR Green ROX qPCR Mastermix (Qiagen; 330521). Gene expression was normalized to the array's *B2M* reference gene and fold change compared to DMSO was calculated using the ΔΔCt method. To validate array data, TaqMan probe based qPCR was carried out using TaqMan Fast Advanced Master Mix (AB; 4444964) and TaqMan primer/probe sets for Sestrin (AB; Hs00902787_m1), TNF (AB; Hs01113624_m1) and GILZ (AB, Hs00608272_m1). Fold change was calculated as above, normalizing to *ACTB* (AB; 4333762F), *B2M* (AB; 4333766F), and *GAPDH* (AB; 4333764F) as reference genes. The average fold change and *p* values were calculated using GraphPad Prism.

### 6) Xenograft Studies

Studies in WSU-DLCL2 and SUDHL6 xenograft models were performed at CRL Piedmont. Piedmont specifically complies with the recommendations of the Guide for Care and Use of Laboratory Animals with respect to restraint, husbandry, surgical procedures, feed and fluid regulation, and veterinary care. The animal program at Piedmont is accredited by the Association for Assessment and Accreditation of Laboratory Animal Care (AAALAC) International, which assures compliance with accepted standards for the care and use of laboratory animals. The protocols were approved by the Institutional Animal Care and Use Committees (IACUC) of CRL Piedmont. All the procedures related to animal handling, care and the treatment in these studies were performed according to the guidelines approved by the IACUC at Piedmont. Studies in the SUDHL10 xenograft models were performed at Shanghai ChemPartner. Shanghai ChemPartner specifically complies with the recommendations of the Guide for Care and Use of Laboratory Animals with respect to restraint, husbandry, surgical procedures, feed and fluid regulation, and veterinary care. The animal program at Shanghai ChemPartner is accredited by the Association for Assessment and Accreditation of Laboratory Animal Care (AAALAC) International, which assures compliance with accepted standards for the care and use of laboratory animals. The protocols were approved by the Institutional Animal Care and Use Committees (IACUC) of Shanghai ChemPartner. All the procedures related to animal handling, care and the treatment in these studies were performed according to the guidelines approved by the IACUC at Shanghai Chempartner. For all studies, mice were closely monitored for overall health status daily, their body weights and tumor volumes were monitored twice a week, and mice were humanly euthanized by carbon dioxide inhalation if their body weights dropped more than 20% of the original weight, or their xenograft tumor grew larger than 2000 mm^3^, or at pre-specified study endpoints. Mice were housed in cages with corn cob/Enrich-o'cobs bedding material, with no more than 5 mice per cage, in rooms at 20–26°C, 40–70% humidity, and a 12-hour light cycle. Food and water were available *ad libitum*.

WSU-DLCL2, SUDHL6, or SUDHL10 cells were harvested during mid-log phase growth, and re-suspended in PBS with 50% Matrigel™ (BD Biosciences), and injected into immune-compromised mice. Female CB17/SCID mice (6–8 weeks in age, ranging from 16–22 g at start of study) were used for the WSU-DLCL2 and SUDHL10 models, sourced from Charles River Laboratories or Beijing Vitalriver Laboratory Animal Co., LTD, respectively. Female athymic nude mice (10 weeks in age, ranging from 18–29 g at start of study) were used for the SUDHL6 model, sourced from Charles River Laboratories. Each mouse received 1×10^7^ cells (0.2 mL cell suspension) subcutaneously in the right flank. Once tumors reached a predetermined size as determined from model development, mice were randomized into groups so that mean tumor volumes were similar in each group. Number of mice per group and number of groups are listed in S1 File tables F–I. Mice were orally dosed with different doses of EPZ-6438 (formulation described previously; [42]) at various schedules for up to 28 days and/or CHOP/COP on the following schedules: Cyclophosphamide (in saline) was administered intraperitoneally (30 mg/kg; i.p.), and Doxorubicin (2.475 mg/kg) and Vincristine (0.375 mg/kg), both in saline, were each administered via bolus tail vein injections (i.v.); each was given once daily on days 1 and 8 in the SUDHL6 study, and on days 1 and 22 in the WSU-DLCL2 and SUDHL10 studies. Prednisone (in saline) was administered orally (0.15 mg/kg, p.o.) on two cycles of five once daily (QD) doses, starting on days 1 and 8 ([QD×5]×2, days 1, 8) in the SUDHL6 study, and on Days 1 and 22 ([QD×5)×2, days 1, 22) in the WSU-DLCL2 and SUDHL10 studies. In the second SUDHL10 study Prednisone was also administered at 0.15 mg/kg QD×28. Each dose was delivered in a volume of 0.2 mL/20 g mouse (10 mL/kg), and adjusted for the last recorded weight of individual animals. Tumor measurements and body weights were collected twice-weekly for 28 days for all studies. To determine tumor growth delay in the SUDHL10 and SUDHL6 studies, each test animal was euthanized when its neoplasm reached the endpoint volume of 2000 mm^3^ or on the last day of the study (day 60), whichever came first. All single agent treatments were tolerated in the SUDHL10 xenograft study; one mouse from the COP dosed group had to be euthanized on Day 15 due to poor body conditions. Three days after dosing of the second cycle of COP in combination with EPZ-6438, body weight loss and 2 mortalities were observed in this group, and mice were left without treatment for 2 days. The group resumed treatment on Day 27. Additional body weight data for all models are listed in S1 File (figures E and F, panel C).

## Supporting Information

S1 Checklist **ARRIVE checklist.**
(PDF)

S1 File **File includes Supplementary text, Figures A–F, Tables S–I, and Supplementary reference.** Figure A: Glucocorticoid Agonists Enhance Potency of EPZ-6438 in SUDHL10 (*EZH2* Y646F) and SUDHL6 (*EZH2* Y646N) cells. Figure B: Global H3K27 Trimethylation Is Unaffected by Prednisolone or Combination Treatment. Figure C: Global H3K27 Acetylation Is Unaffected by Prednisolone or Combination Treatment. Figure D: Glucocorticoid Receptor Expression Is Not Changed with EPZ-6438/Prednisolone Combination Treatment. Figure E: Percent change in body weight for WSU-DLCL2, SUDHL6, and SUDHL10 (EPZ-6438+COP) studies. Figure F: Efficacy of EPZ-6438/COP or EPZ-6438/Prednisone Combinations in SUDHL10 EZH2 Mutant Xenograft Model. Table A: Summary of IC50 Values to Single Agents in Various Lymphoma Cell Lines (nM). Table B: Summary of Maximum IC50 Shifts for EPZ-6438/GRag Combinations in Various GCB Lymphoma Cell Lines. Table C: Summary of Combination Effects with EPZ-6438 in Various GCB Lymphoma Cell Lines. Table D: Results of Cell of Origin Analysis by Hans-Choi Immunohistochemistry. Table E: Statistical Analysis of Glucocorticoid Receptor Gene Expression Presented in S1 File figure D. Tables F–I: Study Design, Including Groups and Number of Mice per Group, for F) WSU-DLCL2, G) SUDHL6, H) SUDHL10 (EPZ-6438+COP), and I) SUDHL10 (EPZ-6438+Prednisone) studies.
(PDF)

S2 File **Ct values from the RT2 glucocorticoid signaling PCR array analysis for 6 GCB DLBCL cell lines.**
(XLS)
