# Supplementary material for: Synergistic Anti-Tumor Activity of EZH2 Inhibitors and Glucocorticoid Receptor Agonists in Models of Germinal Center Non-Hodgkin Lymphomas
Source: PLoS One. 2014 Dec 10;9(12):e111840. doi: 10.1371/journal.pone.0111840 (PMC4262195; doi:10.1371/journal.pone.0111840)
Supplement: S1 File — File includes Supplementary text, Figures A–F, Tables S–I, and Supplementary reference. Figure A: Glucocorticoid Agonists Enhance Potency of EPZ-6438 in SUDHL10 (EZH2 Y646F) and SUDHL6 (EZH2 Y646N) cells. Figure B: Global H3K27 Trimethylation Is Unaffected by Prednisolone or Combination Treatment. Figure C: Global H3K27 Acetylation Is Unaffected by Prednisolone or Combination Treatment. Figure D: Glucocorticoid Receptor Expression Is Not Changed with EPZ-6438/Prednisolone Combination Treatment. Figure E: Percent change in body weight for WSU-DLCL2, SUDHL6, and SUDHL10 (EPZ-6438+COP) studies. Figure F: Efficacy of EPZ-6438/COP or EPZ-6438/Prednisone Combinations in SUDHL10 EZH2 Mutant Xenograft Model. Table A: Summary of IC50 Values to Single Agents in Various Lymphoma Cell Lines (nM). Table B: Summary of Maximum IC50 Shifts for EPZ-6438/GRag Combinations in Various GCB Lymphoma Cell Lines. Table C: Summary of Combination Effects with EPZ-6438 in Various GCB Lymphoma Cell Lines. Table D: Results of Cell of Origin Analysis by Hans-Choi Immunohistochemistry. Table E: Statistical Analysis of Glucocorticoid Receptor Gene Expression Presented in S1 File figure D. Tables F–I: Study Design, Including Groups and Number of Mice per Group, for F) WSU-DLCL2, G) SUDHL6, H) SUDHL10 (EPZ-6438+COP), and I) SUDHL10 (EPZ-6438+Prednisone) studies. (PDF) [file pone.0111840.s002.pdf]

Supplementary Text

When the anti-proliferative activity of a compound is affected by a second compound (A) of distinct mechanism of action, the effect of varying concentration of A ([A]) on the IC_{50} of the first compound can be described by the following relationship [1].

$$IC_{50}{}^{[A]} = \frac{[A] + K_A}{\frac{K_A}{IC_{50}{}^0} + \frac{[A]}{\alpha IC_{50}{}^0}} \quad \text{(A)}$$

where [A] and K_A are the concentration of compound A and its single agent anti-proliferative IC_{50} for the cell line under study (which may not be experimentally determinable), respectively. $IC_{50}{}^{[A]}$ is the IC_{50} of the test compound in combination with [A] and $IC_{50}{}^0$ is the IC_{50} of the test compound alone (i.e., at [A] = 0). The term α is a constant that indicates the degree of potency enhancement effected by compound A and is given by the following ratio.

$$\alpha = \frac{IC_{50}{}^{\infty}}{IC_{50}{}^0} \quad \text{(B)}$$

$IC_{50}{}^{\infty}$ is the limit of the IC_{50} value of the test compound expected at infinite concentration of compound A. Thus, the reciprocal of α gives the maximum fold-shift in IC_{50} effected by compound A.

$$\frac{1}{\alpha} = \frac{{IC_{50}}^{0}}{{IC_{50}}^{\infty}} \quad \text{(C)}$$

Supplementary File S1 Figure and Table Legends

Figure A. Glucocorticoid Agonists Enhance Potency of EPZ-6438 in SUDHL10 (*EZH2* Y646F) and SUDHL6 (*EZH2* Y646N) cells

Combinations of EPZ-6438 and Prednisolone or Dexamethasone in SUDHL10 (**A, B**) and SUDHL6 (**C, D**) GCB cell lines, respectively. All dose response plots were generated in Graphpad Prism and curves fitted to a four-parameter model with variable slope. The IC_{50} values at varying concentrations of Prednisolone or Dexamethasone were fitted to equation S2 to determine α. **A, B)** Potency of EPZ-6438 was increased a maximum of 2.6-fold with Prednisolone and 5.6-fold with Dexamethasone in the SUDHL10 cell line. **C, D)** Potency of EPZ-6438 was increased a maximum of 55.5-fold with Prednisolone and 250-fold with Dexamethasone in the SUDHL6 cell line. Doses of EPZ-6438 ranged from 1.56-100 nM, doses of Prednisolone ranged from 7.8-1000 nM and doses of Dexamethasone ranged from 1.6-200 nM.

Figure B. Global H3K27 Trimethylation Is Unaffected by Prednisolone or Combination Treatment

Cells were treated for 4 days with increasing doses of Prednisolone, EPZ-6438, or a combination of EPZ-6438 with a constant dose of Prednisolone. Acid extracted histones were analyzed by ELISA for H3K27Me3 levels (Prednisolone alone, left panel; EPZ-6438/Prednisolone combination, right panel, with IC_{50} values as insets of each graph). For Prednisolone treatment, H3K27Me3 values are represented as a bar graph. Data are presented as the mean of two biological replicates. Error bars represent SD values. Cpd = Compound.

Figure C. Global H3K27 Acetylation Is Unaffected by Prednisolone or Combination Treatment

WSU-DLCL2 (**A**), OCI-LY19 (**B**) or RL cells (**C**) were treated for 4 days with increasing doses of Prednisolone, EPZ-6438, or a combination of EPZ-6438 with a constant dose of Prednisolone. Acid extracted histones were analyzed by western blot for H3K27 acetylation levels. Blots were quantified by densitometry, and data are presented as the mean of 2 biological replicates in bar graphs (one of the replicates is presented as western blot). Error bars represent SD values.

Figure D: Glucocorticoid Receptor Expression Is Not Changed with EPZ-6438/Prednisolone Combination Treatment

Expression levels of glucocorticoid receptor, normalized to DMSO controls, for each cell line treated with the indicated single agents or their combination (2 biological replicates, see methods materials and methods section 5 for details). Fold change values were quantified using the $\Delta\Delta$Ct method and *ACTB*, *B2M* and *GAPDH* as reference genes. Error bars represent SEM values. Statistical analysis of the data is presented in table E.

Figure E. Percent change in body weight for WSU-DLCL2, SUDHL6, and SUDHL10 (EPZ-6438+COP) studies.

Percent body weight change in **A)** WSU-DLCL2 study, **B)** SUDHL6 study, and **C)** first SUDHL10 (EPZ-6438+COP) study, as described in figure 5.

CHOP: Cyclophosphamide, Hydroxyldaunomycin (Doxorubicin), Oncovin (Vincristine) and Prednisone; COP: Cyclophosphamide, Oncovin (Vincristine) and Prednisone; BID: two times a day every 12 hours; QD: once a day; TID: three times a day every 8 hours.

Figure F. Efficacy of EPZ-6438/COP or EPZ-6438/Prednisone Combinations in SUDHL10 *EZH2* Mutant Xenograft Model

A) SUDHL10 (*EZH2* Y646F) xenograft-bearing mice were treated with EPZ-6438, COP (chemotherapy without the Doxorubicin component), or their combination for 28 days, as specified in the methods. Mean tumor weights from 8/16 mice, euthanized on day 28, are compared, demonstrating the significant differences in tumor weight between groups (* $p < 0.05$, ** $p < 0.01$, **** $p < 0.0001$; two-tailed *t* test). **B)** SUDHL10 (*EZH2* Y646F) xenograft-bearing mice were treated for 28 days with two doses of EPZ-6438 or Prednisone at two different schedules (Pred-1 = Prednisone at 0.15 mg/kg BID x 5 on days 1-5 and 22-26; Pred-2 = Prednisone 0.15 mg/kg BID x 28). Both compounds were also administered in combination as indicated. Mean tumor volumes ± SEM (n=10) are plotted in top panel. All groups administered EPZ-6438 show statistically significant reduction in tumor growth ($p < 0.01$ at least, vs. vehicle or Prednisone single agent at both schedules; repeated measures ANOVA, Dunnett's post test),

while Prednisone single agent did not elicit any significant anti-tumor effect compared to vehicle. **C)** Matching body weight graph for panel B.

BID: two times a day every 12 hours; QD: once a day.

Table A. Summary of IC_{50} Values to Single Agents in Various Lymphoma Cell Lines (nM)

Table B. Summary of Maximum IC_{50} Shifts for EPZ-6438/GRag Combinations in Various GCB Lymphoma Cell Lines

Table C. Summary of Combination Effects with EPZ-6438 in Various GCB Lymphoma Cell Lines

Table D. Results of Cell of Origin Analysis by Hans-Choi Immunohistochemistry

Table E: Statistical Analysis of Glucocorticoid Receptor Gene Expression Presented in Supplementary File S1 Figure D

Tables F-I. Study Design, Including Groups and Number of Mice per Group, for F) WSU-DLCL2, G) SUDHL6, H) SUDHL10 (EPZ-6438+COP), and I) SUDHL10 (EPZ-6438+Prednisone) studies

Supplementary reference

1. Copeland RA (2013) Evaluation of Enzyme Inhibitors in Drug Discovery: A Guide for Medicinal Chemists and Pharmacologists, 2nd Ed.: Wiley, Hoboken, NJ.

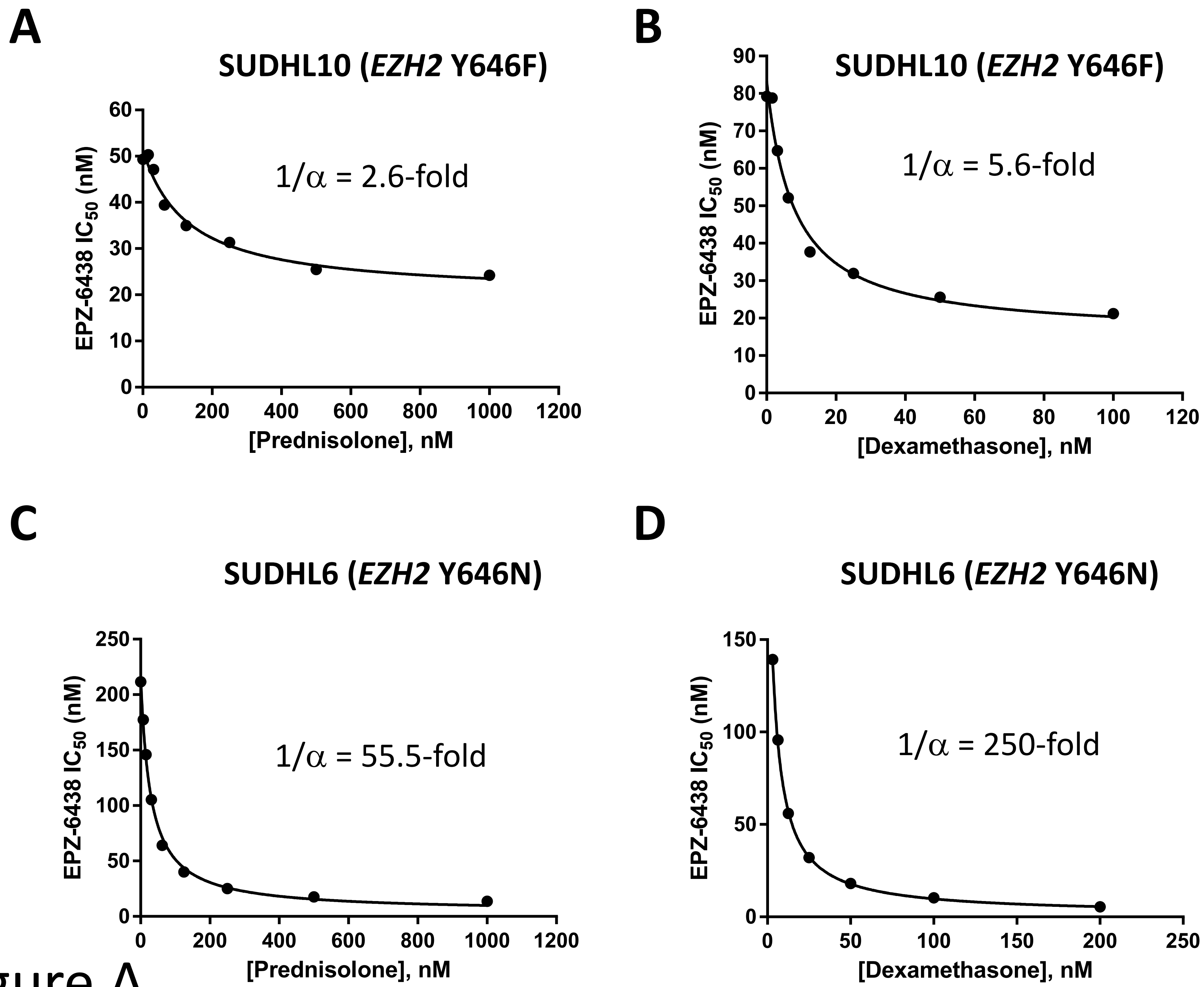
A
SUDHL10 (EZH2 Y646F)
1/α = 2.6-fold
EPZ-6438 IC50 (nM)
[Prednisolone], nM
0
10
20
30
40
50
60
0
200
400
600
800
1000
1200
B
SUDHL10 (EZH2 Y646F)
1/α = 5.6-fold
EPZ-6438 IC50 (nM)
[Dexamethasone], nM
0
10
20
30
40
50
60
70
80
90
0
20
40
60
80
100
120
C
SUDHL6 (EZH2 Y646N)
1/α = 55.5-fold
EPZ-6438 IC50 (nM)
[Prednisolone], nM
0
50
100
150
200
250
0
200
400
600
800
1000
1200
D
SUDHL6 (EZH2 Y646N)
1/α = 250-fold
EPZ-6438 IC50 (nM)
[Dexamethasone], nM
0
50
100
150
0
50
100
150
200
250

Figure A

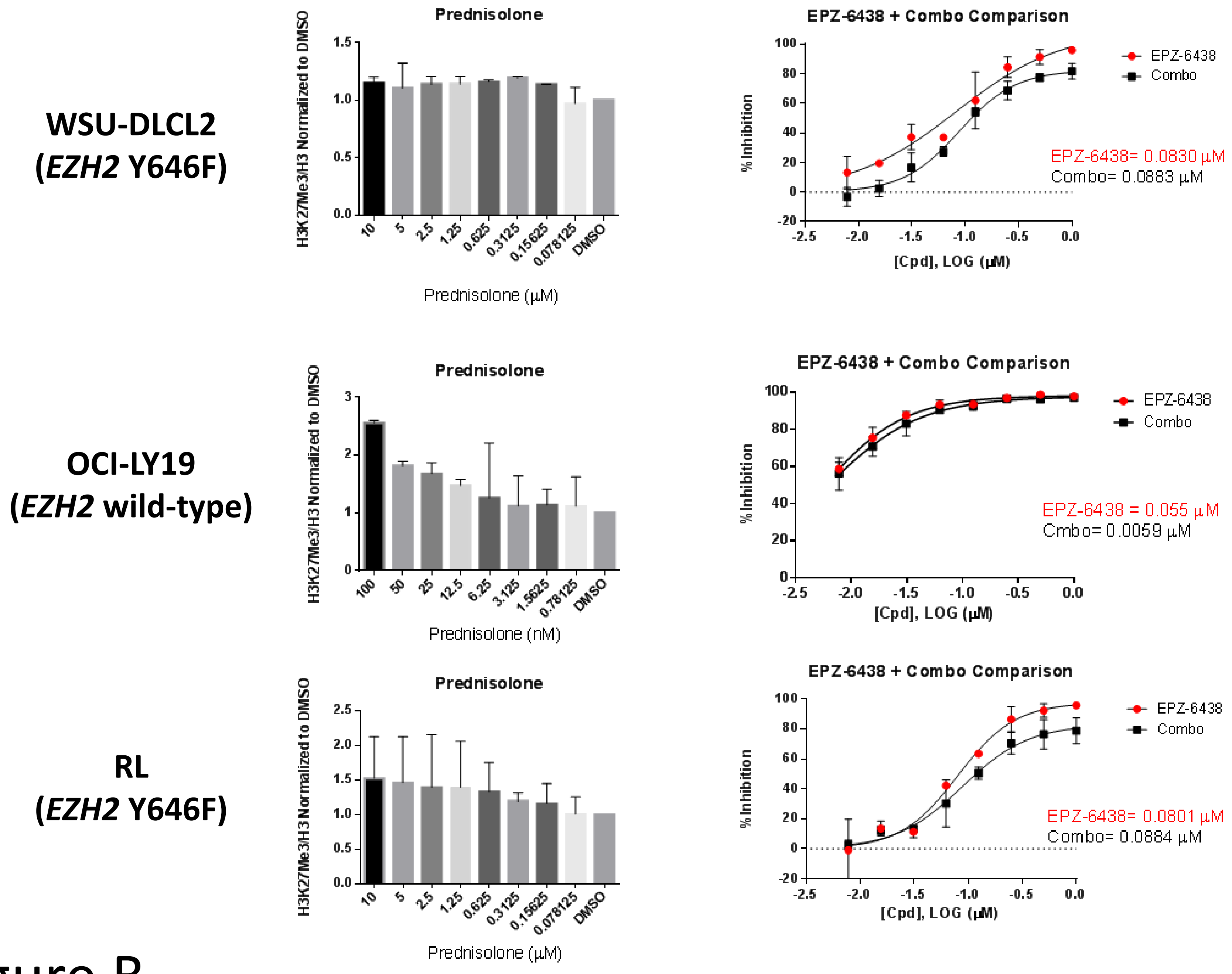

WSU-DLCL2
(EZH2 Y646F)
Prednisolone
H3K27Me3/H3 Normalized to DMSO
1.5
1.0
0.5
0.0
10
5
2.5
1.25
0.625
0.3125
0.15625
0.078125
DMSO
Prednisolone (µM)
EPZ-6438 + Combo Comparison
% Inhibition
100
80
60
40
20
0
-20
-2.5
-2.0
-1.5
-1.0
-0.5
0.0
[Cpd], LOG (µM)
EPZ-6438
Combo
EPZ-6438= 0.0830 µM
Combo= 0.0883 µM
OCI-LY19
(EZH2 wild-type)
Prednisolone
H3K27Me3/H3 Normalized to DMSO
3
2
1
0
100
50
25
12.5
6.25
3.125
1.5625
0.78125
DMSO
Prednisolone (nM)
EPZ-6438 + Combo Comparison
% Inhibition
100
80
60
40
20
0
-2.5
-2.0
-1.5
-1.0
-0.5
0.0
[Cpd], LOG (µM)
EPZ-6438
Combo
EPZ-6438 = 0.055 µM
Cmbo= 0.0059 µM
RL
(EZH2 Y646F)
Prednisolone
H3K27Me3/H3 Normalized to DMSO
2.5
2.0
1.5
1.0
0.5
0.0
10
5
2.5
1.25
0.625
0.3125
0.15625
0.078125
DMSO
Prednisolone (µM)
EPZ-6438 + Combo Comparison
% Inhibition
100
80
60
40
20
0
-20
-2.5
-2.0
-1.5
-1.0
-0.5
0.0
[Cpd], LOG (µM)
EPZ-6438
Combo
EPZ-6438= 0.0801 µM
Combo= 0.0884 µM

Figure B

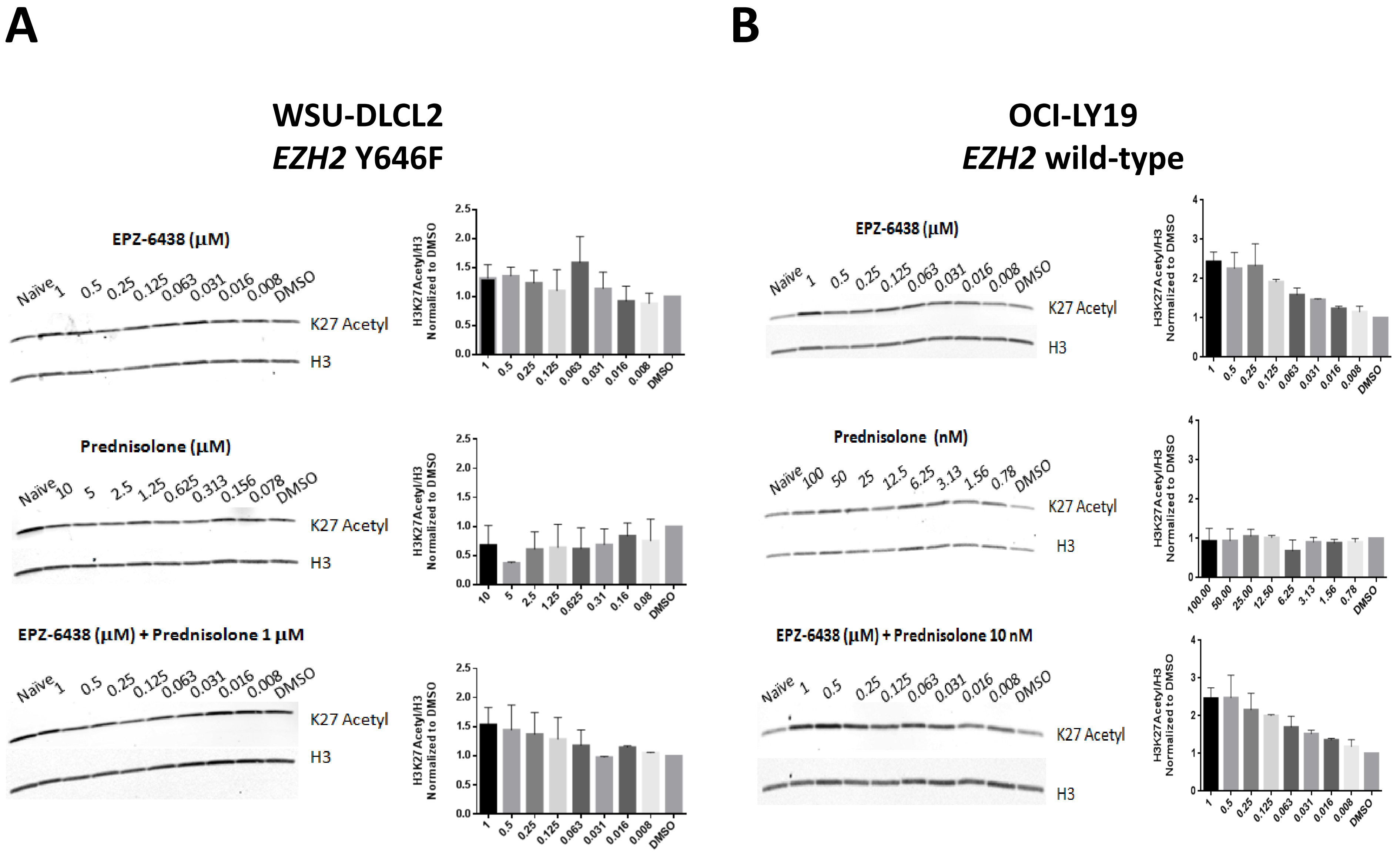

Figure C, panels A and B

C

RL
***EZH2* Y646N**

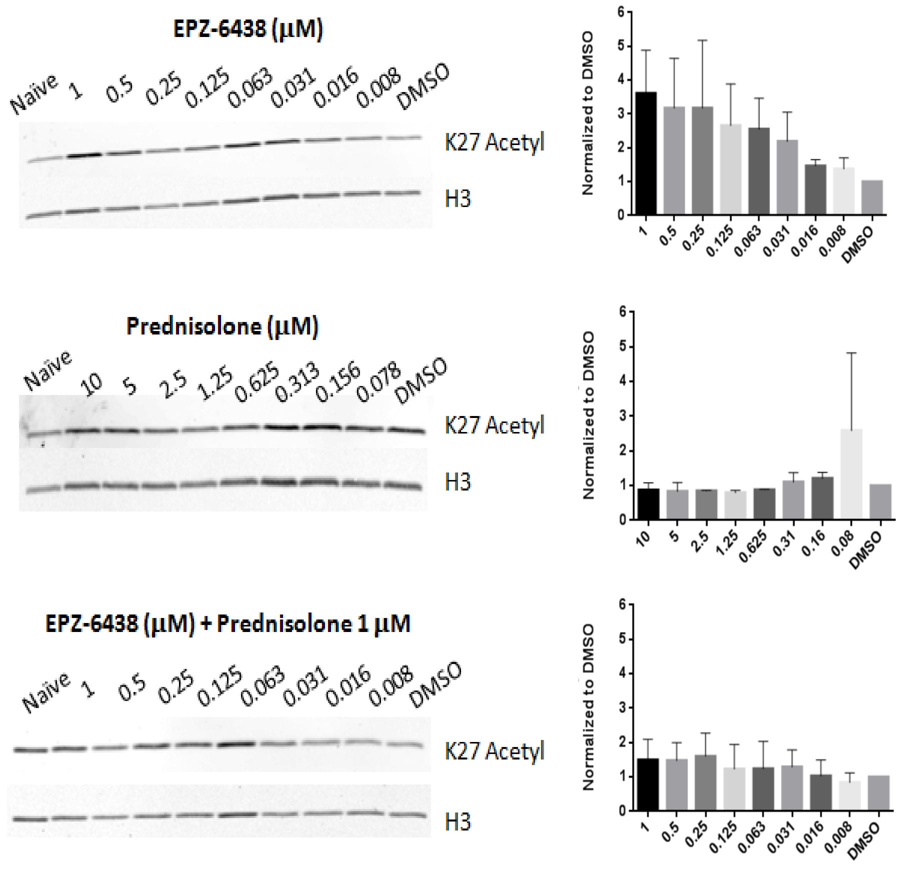

Figure C, panel C

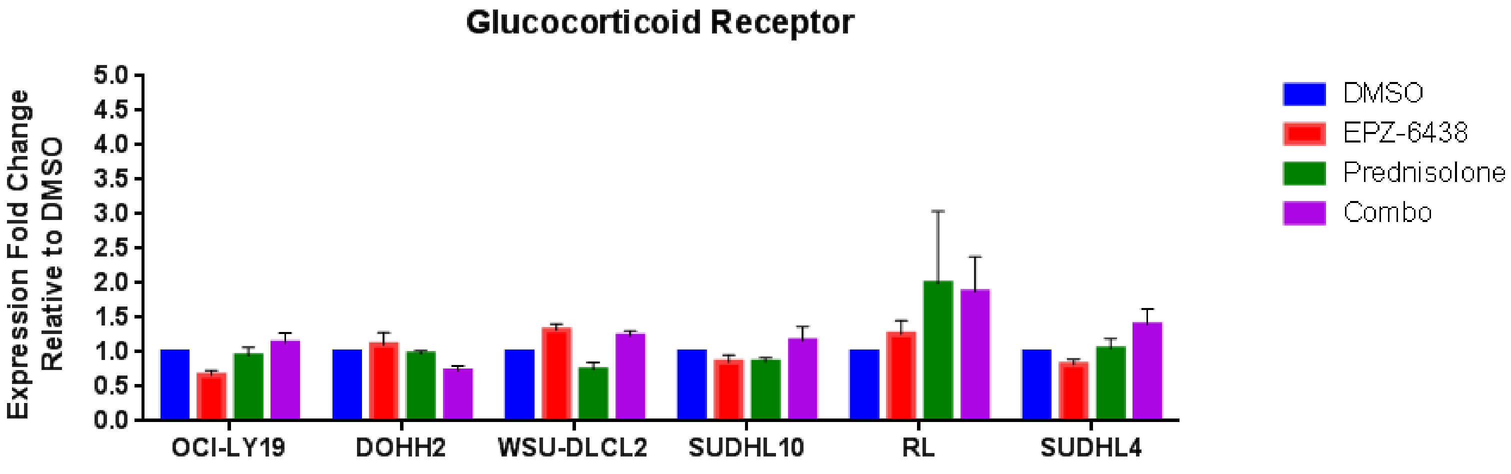

EZH2 WT

EZH2 Y646-Sensitive

EZH2 Y646-Resistant

Figure D

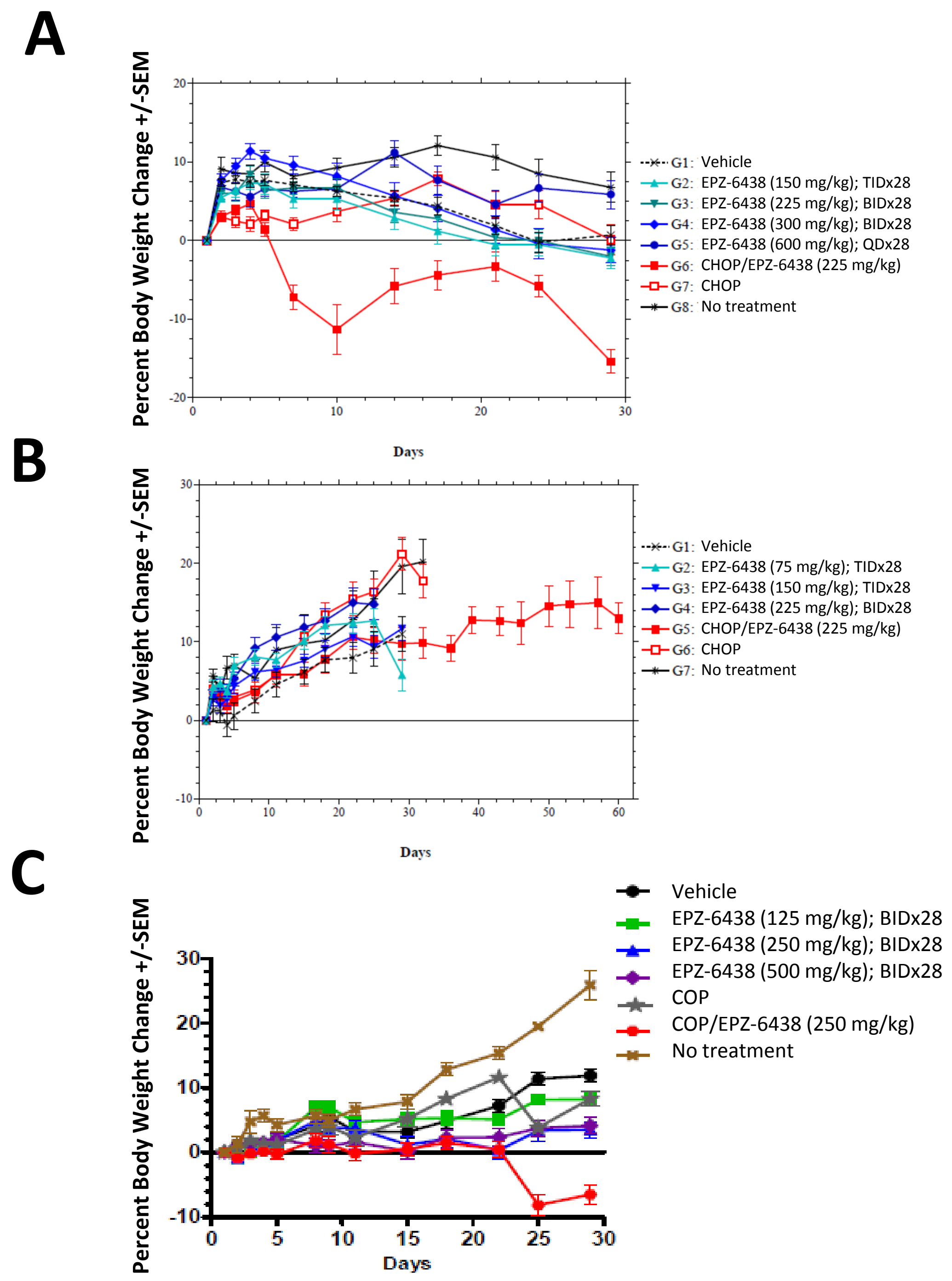
A
Percent Body Weight Change +/-SEM
Days
G1: Vehicle
G2: EPZ-6438 (150 mg/kg); TIDx28
G3: EPZ-6438 (225 mg/kg); BIDx28
G4: EPZ-6438 (300 mg/kg); BIDx28
G5: EPZ-6438 (600 mg/kg); QDx28
G6: CHOP/EPZ-6438 (225 mg/kg)
G7: CHOP
G8: No treatment
B
Percent Body Weight Change +/-SEM
Days
G1: Vehicle
G2: EPZ-6438 (75 mg/kg); TIDx28
G3: EPZ-6438 (150 mg/kg); TIDx28
G4: EPZ-6438 (225 mg/kg); BIDx28
G5: CHOP/EPZ-6438 (225 mg/kg)
G6: CHOP
G7: No treatment
C
Percent Body Weight Change +/-SEM
Days
Vehicle
EPZ-6438 (125 mg/kg); BIDx28
EPZ-6438 (250 mg/kg); BIDx28
EPZ-6438 (500 mg/kg); BIDx28
COP
COP/EPZ-6438 (250 mg/kg)
No treatment

Figure E

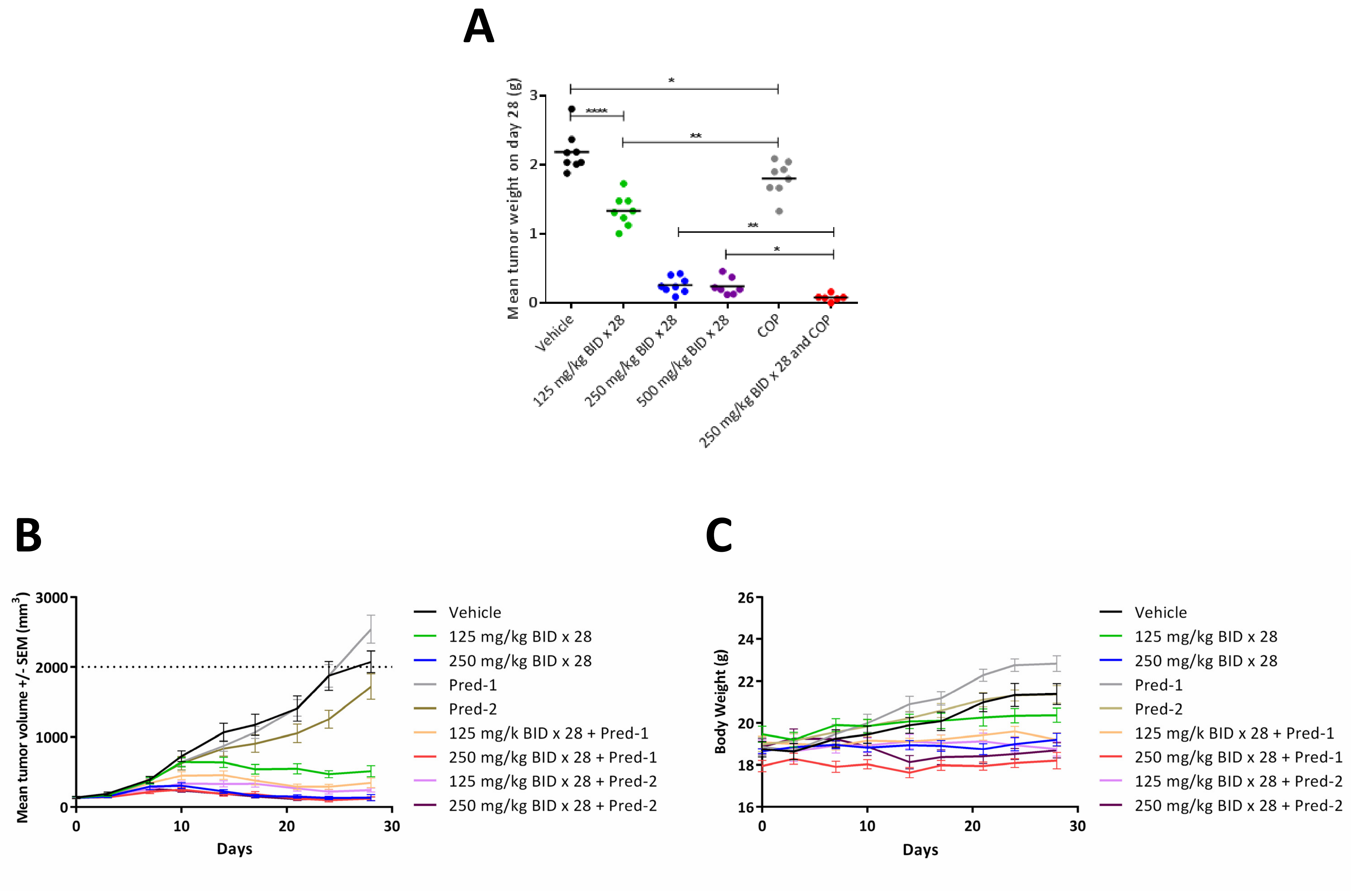

A
Mean tumor weight on day 28 (g)
3
2
1
0
*
****
**
**
*
Vehicle
125 mg/kg BID x 28
250 mg/kg BID x 28
500 mg/kg BID x 28
COP
250 mg/kg BID x 28 and COP
B
Mean tumor volume +/- SEM (mm3)
3000
2000
1000
0
0
10
20
30
Days
Vehicle
125 mg/kg BID x 28
250 mg/kg BID x 28
Pred-1
Pred-2
125 mg/k BID x 28 + Pred-1
250 mg/kg BID x 28 + Pred-1
125 mg/kg BID x 28 + Pred-2
250 mg/kg BID x 28 + Pred-2
C
Body Weight (g)
26
24
22
20
18
16
0
10
20
30
Days
Vehicle
125 mg/kg BID x 28
250 mg/kg BID x 28
Pred-1
Pred-2
125 mg/k BID x 28 + Pred-1
250 mg/kg BID x 28 + Pred-1
125 mg/kg BID x 28 + Pred-2
250 mg/kg BID x 28 + Pred-2

Figure F

Table A: Summary of IC_{50} Values to Single Agents in Various GCB Lymphoma Cell Lines (nM)

Cell Line	*EZH2* Status	EPZ-6438 Day 4	Prednisolone Day 3	Doxorubicin Day 3	Mafosfamide Day 3	Vincristine Day 3	Dexamethasone Day 3
WSU-DLCL2	Y646F GCB	530	>1000	8	2000	1	>1000
SUDHL10	Y646F GCB	640	>1000	6	1200	0.5	>1000
SUDHL6	Y646N GCB	220[a]	>1000	18	3000	0.2	350[b]
RL	Y646N GCB	>1000	>1000	ND	ND	ND	ND
SUDHL4	Y646S GCB	>1000	>1000	ND	ND	ND	ND
DOHH2	Wild-type GCB	>1000	1300	2	588	0.5	100
OCI-LY19	Wild-type GCB	>1000	59	3	335	0.6	9
SUDHL5	Wild-type GCB	>1000	39	ND	ND	ND	5
Toledo[c]	Wild-type GCB	>1000	ND[d]	8	880	2	ND[d]

a: EPZ-6438 IC_{50} value for SUDHL6 cells determined on day 7.
b: Plateau at 55% inhibition.
c: IC_{50} values of Toledo cells were determined on day 5.
d: IC_{50} calculation not possible; dose response curve did not fit the 4-paramenter logistic model.
GCB: germinal center B cell-derived; ND: not determined

Table B: Summary of Maximum IC_{50} Shifts for EPZ-6438/GRag Combinations in Various GCB Lymphoma Cell Lines

Cell Line	*EZH2* Status	Varied Compound	Effector Compound	IC_{50}^{0}, nM[a, b]	IC_{50}^{∞}, nM[c, d]	α	1/α (IC_{50}^{0}/ IC_{50}^{∞})
WSU-DLCL2	Y646F GCB	EPZ-6438	Prednisolone	100.02	4.12	0.04 ± 0.035	24
WSU-DLCL2	Y646F GCB	EPZ-6438	Dexamethasone	283.1	9.34	0.03 ± 0.027	30
SUDHL10	Y646F GCB	EPZ-6438	Prednisolone	51.4	19.53	0.38 ± 0.04	2.6
SUDHL10	Y646F GCB	EPZ-6438	Dexamethasone	83.3	15.3	0.18 ± 0.05	5.6
SUDHL6	Y646N GCB	EPZ-6438	Prednisolone	216.3	3.89	0.018 ± 0.017	55.6
SUDHL6	Y646N GCB	EPZ-6438	Dexamethasone	272.1	1.09	0.004 ± 0.002	250
DOHH2	Wild-type GCB	Prednisolone	EPZ-6438	331.8	35.1	0.106 ± 0.016	9.5
DOHH2	Wild-type GCB	Dexamethasone	EPZ-6438	91.4	10.9	0.12 ± 0.007	8.3
SUDHL5	Wild-type GCB	Prednisolone	EZP-6438	510.4	64.2	0.126 ± 0.027	7.9
SUDHL5	Wild-type GCB	Dexamethasone	EPZ-6438	3.79	0.28	0.07 ± 0.039	14.3
OCI-LY19	Wild-type GCB	Prednisolone	EPZ-6438	44.7	7.47	0.17 ± 0.06	5.9
OCI-LY19	Wild-type GCB	Dexamethasone	EPZ-6438	6.2	0.92	0.13 ± 0.06	7.7
Toledo	Wild-type GCB	Prednisolone	EPZ-6438	ND[e]	NA[f]	ND[g]	ND[g]
Toledo	Wild-type GCB	Dexamethasone	EPZ-6438	ND[e]	NA[f]	ND[g]	ND[g]

a: Derived after incubation for 7 days for EPZ-6438 in the absence of Prednisolone or Dexamethasone.
b: Derived after incubation for 3 days for Prednisolone or Dexamethasone in the absence of EPZ-6438.
c: Derived after incubation for 7 days for EPZ-6438 assuming an infinite concentration of Prednisolone or Dexamethasone.
d: Derived after incubation for 3 days for Prednisolone or Dexamethasone assuming an infinite concentration of EPZ-6438.
e: IC_{50} calculation not possible; dose response curve did not fit the 4-paramenter logistic model.
f: Sensitization was not observed at any dose of EPZ-6438.
g: IC_{50}^{0} not determined, calculation of α not possible
GCB: germinal center B cell-derived; GRag: glucocorticoid receptor agonist; NA: not available; ND: not determined

Table C: Summary of Combination Effects With EPZ-6438 in Various GCB Lymphoma Cell Lines

Treatment		WSU-DLCL2 *EZH2* Y646F GCB	SUDHL10 *EZH2* Y646F GCB	SUDHL6 *EZH2* Y646N GCB	DOHH2 *EZH2* wild-type GCB	SUDHL5 *EZH2* wild-type GCB	OCI-LY19 *EZH2* wild-type GCB	Toledo *EZH2* wild-type GCB
Standard of Care	Prednisolone	Potency enhanced 7x[a]	Potency enhanced 3x[a]	Potency enhanced 11x[a]	Potency enhanced 2x[b]	Potency enhanced 6x[c]	Potency enhanced 12x[b]	No effect[d]
	Doxorubicine	Synergy	Additive	Additive	No effect	ND	ND	No effect
	Mafosfamide	Additive	Additive	Additive	No effect	ND	ND	No effect
	Vincristine	Additive	Additive	Additive	No effect	ND	ND	No effect
Other Therapies	Dexamethasone	Potency enhanced 15x[e]	Potency enhanced 5x[e]	Potency enhanced 18x[f]	Potency enhanced 4x[g]	Potency enhanced 8x[h]	Potency enhanced 16x[i]	No effect[j]

a: Shift for EPZ-6438 IC_{50} value at 1 µM Prednisolone
b: Shift for Prednisolone IC_{50} value at 2 µM EPZ-6438
c: Shift for Prednisolone IC_{50} value at 5 µM EPZ-6438
d: Sensitization of Prednisolone effect was not observed at 1 µM EPZ-6438
e: Shift for EPZ-6438 IC_{50} value at 0.1 µM Dexamethasone
f: Shift for EPZ-6438 IC_{50} value at 0.2 µM Dexamethasone
g: Shift for Dexamethasone IC_{50} at 10 µM EPZ-6438
h: Shift for Dexamethasone IC_{50} value at 5 µM EPZ-6438
i: Shift for Dexamethasone IC_{50} value at 2 µM EPZ-6438
j: Sensitization of Dexamethasone effect was not observed at 1 µM EPZ-6438
GCB: germinal center B cell-derived; ND: not determined

Table D: Results of Cell of Origin Analysis by Hans-Choi Immunohistochemistry

Cell Line	*EZH2* Status	GCET1	MUM1	CD10	BCL6	FOXP1	Hans Score	Choi Score	Comments
WSU-DLCL2	Y646F GCB	50	0	90	100	80	GCB	GCB	Low level positive of FOXP1
SUDHL4	Y646S GCB	50	0	100	100	70	GCB	GCB	
SUDHL6	Y646N GCB[e]	50	20	40	100	80	GCB	GCB	Many weakly positive FOXP1 cells
SUDHL10	Y646F GCB	80	0	100	100	50	GCB	GCB	Many weak forms of FOXP1 cells
RL	Y646N GCB	90	0	100	100	100	GCB	GCB	Variable intensity FOXP1
DOHH2	Wild-type GCB	50	0	100	100	100	GCB	GCB	
OCI-LY19	Wild-type GCB	0	30	70	50	100	GCB	GCB	Very rare GCET1 positive cells, weak MUM1 positivity, variable BCL6 positivity
SUDHL5	Wild-type GCB	90	0	100	100	100	GCB	GCB	
Toledo	Wild-type GCB	0	90	100	10	100	GCB	GCB	Very rare GCET1 positive cells

Numbers in the table represent % positive cells in a given specimen
GCB: Germinal center B cell-derived

Table E: Statistical Analysis of Glucocorticoid Receptor Gene Expression Presented in Figure S4

Cell Line	Comparison	*Glucocorticoid Receptor*	
		***P* Value**	***P* Value Summary**
OCI-LY19	DMSO vs Combo	0.3878	ns
OCI-LY19	EPZ-6438 vs Combo	0.0408	*
OCI-LY19	Prednisolone vs Combo	0.3666	ns
DOHH2	DMSO vs Combo	0.0202	*
DOHH2	EPZ-6438 vs Combo	0.1151	ns
DOHH2	Prednisolone vs Combo	0.0489	*
WSU-DLCL2	DMSO vs Combo	0.0302	*
WSU-DLCL2	EPZ-6438 vs Combo	0.5117	ns
WSU-DLCL2	Prednisolone vs Combo	0.0185	*
SUDHL10	DMSO vs Combo	0.4688	ns
SUDHL10	EPZ-6438 vs Combo	0.2508	ns
SUDHL10	Prednisolone vs Combo	0.2305	ns
RL	DMSO vs Combo	0.1613	ns
RL	EPZ-6438 vs Combo	0.3253	ns
RL	Prednisolone vs Combo	0.9231	ns
SUDHL4	DMSO vs Combo	0.1589	ns
SUDHL4	EPZ-6438 vs Combo	0.0754	ns
SUDHL4	Prednisolone vs Combo	0.2735	ns

Pairwise statistical comparisons were performed by two-tailed *t* test.
ns: not significant; * $p < 0.05$

Table F: Study Design for the WSU-DLCL2 Xenograft Model

Group	n	Treatment Regimen 1				Treatment Regimen 2				Treatment Regimen 3				Treatment Regimen 4				Treatment Regimen 5			
		Agent	mg/kg	Route	Schedule	Agent	mg/kg	Route	Schedule	Agent	mg/kg	Route	Schedule	Agent	mg/kg	Route	Schedule	Agent	mg/kg	Route	Schedule
1	12	V	-	po	BIDx28	-	-	-	-	-	-	-	-	-	-	-	-	-	-	-	-
2	12	EM10	150	po	TIDx28	-	-	-	-	-	-	-	-	-	-	-	-	-	-	-	-
3	12	EM10	225	po	BIDx28	-	-	-	-	-	-	-	-	-	-	-	-	-	-	-	-
4	12	EM10	300	po	BIDx28	-	-	-	-	-	-	-	-	-	-	-	-	-	-	-	-
5	12	EM10	600	po	QDx28	-	-	-	-	-	-	-	-	-	-	-	-	-	-	-	-
6	12	C	30	ip	Days 1, 22	D	2.48	iv	Days 1, 22	VC	0.38	iv	Days 1, 22	P	0.15	po	(QDx5)x2; Days 1, 22	EM10	225	po	BIDx28
7	12	C	30	ip	Days 1, 22	D	2.48	iv	Days 1, 22	VC	0.38	iv	Days 1, 22	P	0.15	po	(QDx5)x2; Days 1, 22	-	-	-	-
8	8	NT	-	-	-	NT	-	-	-	NT	-	-	-	NT	-	-	-	NT	-	-	-

BID: twice a day every 12 h; C: Cyclophosphamide; D: Doxorubicin; EM10: EPZ-6438; ip: intraperitoneal; iv: intravenously; NT: no treatment; P: Prednisone; po: by oral gavage; QD: once a day; TID: three times a day every 8 h; V: vehicle; VC: Vincristine

Table G: Study Design for the SUDHL6 Xenograft Model

Group	n	Treatment Regimen 1				Treatment Regimen 2				Treatment Regimen 3				Treatment Regimen 4				Treatment Regimen 5			
		Agent	mg/kg	Route	Schedule	Agent	mg/kg	Route	Schedule	Agent	mg/kg	Route	Schedule	Agent	mg/kg	Route	Schedule	Agent	mg/kg	Route	Schedule
1	12	V	-	po	BIDx28	-	-	-	-	-	-	-	-	-	-	-	-	-	-	-	-
2	12	EM10	75	po	TIDx28	-	-	-	-	-	-	-	-	-	-	-	-	-	-	-	-
3	12	EM10	150	po	TIDx28	-	-	-	-	-	-	-	-	-	-	-	-	-	-	-	-
4	12	EM10	225	po	BIDx28	-	-	-	-	-	-	-	-	-	-	-	-	-	-	-	-
5	12	C	30	ip	Days 1, 8	D	2.48	iv	Days 1, 8	VC	0.38	iv	Days 1, 8	P	0.15	-	QDx5)x2; Days 1, 8	EM10	225	po	BIDx28
6	12	C	30	ip	Days 1, 8	D	2.48	iv	Days 1, 8	VC	0.38	iv	Days 1, 8	P	0.15	po	(QDx5)x2; Days 1, 8	-	-	-	-
7	8	NT	-	-	-	NT	-	-	-	NT	-	-	-	NT	-	-	-	NT	-	-	-

BID: twice a day every 12 h; C: Cyclophosphamide; D: Doxorubicin; EM10: EPZ-6438; ip: intraperitoneal; iv: intravenously; NT: no treatment; P: Prednisone; po: by oral gavage; QD: once a day; TID: three times a day every 8 h; V: vehicle; VC: Vincristine

Table H: Study Design for the SUDHL10 Xenograft Model (EPZ-6438+COP)

Group	n	Treatment Regimen 1				Treatment Regimen 2				Treatment Regimen 3				Treatment Regimen 4			
		Agent	mg/kg	Route	Schedule	Agent	mg/kg	Route	Schedule	Agent	mg/kg	Route	Schedule	Agent	mg/kg	Route	Schedule
1	16	V	-	po	BIDx28	-	-	-	-	-	-	-	-	-	-	-	-
2	16	EM10	125	po	TIDx28	-	-	-	-	-	-	-	-	-	-	-	-
3	16	EM10	250	po	TIDx28	-	-	-	-	-	-	-	-	-	-	-	-
4	16	EM10	500	po	BIDx28	-	-	-	-	-	-	-	-	-	-	-	-
5	16	C	30	ip	Days 1, 22	VC	0.38	iv	Days 1, 22	P	0.15	-	QDx5)x2; Days 1, 22	EM10	-	-	-
6	16	C	30	ip	Days 1, 22	VC	0.38	iv	Days 1, 22	P	0.15	po	(QDx5)x2; Days 1, 22	-	250	po	BIDx28
7	8	NT	-	-	-	NT	-	-	-	NT	-	-	-	NT	-	-	-

BID: twice a day every 12 h; C: Cyclophosphamide; EM10: EPZ-6438; ip: intraperitoneal; iv: intravenously; NT: no treatment; P: Prednisone; po: by oral gavage; QD: once a day; V: vehicle; VC: Vincristine

Table I: Study Design for the SUDHL10 Xenograft Model (EPZ-6438+Prednisone)

Group	N	Treatment	Dose (mg/kg)	Route	Schedule
1	10	Vehicle	-	po	BIDx28
2	10	EPZ-6438	125	po	BIDx28
3	10	EPZ-6438	250	po	BIDx28
4	10	Prednisone	0.15	po	QDx5, 2 cycles on Day 1 and Day 22
5	10	Prednisone	0.15	po	QDx28
6	10	Prednisone	0.15	po	QDx5, 2 cycles on Day 1 and Day 22
		EPZ-6438	125	po	BIDx28
7	10	Prednisone	0.15	po	QDx5, 2 cycles on Day 1 and Day 22
		EPZ-6438	250	po	BIDx28
8	10	Prednisone	0.15	po	QDx28
		EPZ-6438	125	po	BIDx28
9	10	Prednisone-	0.15	po	QDx28
		EPZ-6438	250	po	BIDx28

BID: twice a day every 12 h; po: by oral gavage; QD: once a day
